# What drives and inhibits researchers to share and use open research data? A systematic literature review to analyze factors influencing open research data adoption

**DOI:** 10.1371/journal.pone.0239283

**Published:** 2020-09-18

**Authors:** Anneke Zuiderwijk, Rhythima Shinde, Wei Jeng

**Affiliations:** 1 Faculty of Technology, Policy and Management, Delft University of Technology, Delft, the Netherlands; 2 D-BAUG Ökologisches Systemdesign, ETH Zürich, Zürich, Switzerland; 3 Department of Library and Information Science, National Taiwan University, Taipei, Taiwan; Aalborg University, DENMARK

## Abstract

Both sharing and using open research data have the revolutionary potentials for forwarding scientific advancement. Although previous research gives insight into researchers’ drivers and inhibitors for sharing and using open research data, both these drivers and inhibitors have not yet been integrated via a thematic analysis and a theoretical argument is lacking. This study’s purpose is to systematically review the literature on individual researchers’ drivers and inhibitors for sharing and using open research data. This study systematically analyzed 32 open data studies (published between 2004 and 2019 inclusively) and elicited drivers plus inhibitors for both open research data sharing and use in eleven categories total that are: ‘the researcher’s background’, ‘requirements and formal obligations’, ‘personal drivers and intrinsic motivations’, ‘facilitating conditions’, ‘trust’, ‘expected performance’, ‘social influence and affiliation’, ‘effort’, ‘the researcher’s experience and skills’, ‘legislation and regulation’, and ‘data characteristics.’ This study extensively discusses these categories, along with argues how such categories and factors are connected using a thematic analysis. Also, this study discusses several opportunities for altogether applying, extending, using, and testing theories in open research data studies. With such discussions, an overview of identified categories and factors can be further applied to examine both researchers’ drivers and inhibitors in different research disciplines, such as those with low rates of data sharing and use versus disciplines with high rates of data sharing plus use. What’s more, this study serves as a first vital step towards developing effective incentives for both open data sharing and use behavior.

## Introduction

Both sharing and using open research data have the revolutionary potentials for forwarding scientific advancement [1–4]. Open research data use combined with new Information and Communication Technologies (e.g., new semantic standards, increasing computing power, increasing/cheaper data-storage capacity)–which has shortened geographical, disciplinary, and expertise’s distances–now offers tremendous opportunities [4]. And now researchers worldwide can more efficiently reproduce each other’s research [2], ferret out any possible poor analyses and fraud [5], make novel scientific discoveries [6], and thus overall work more efficiently [7].

Previous research already provides insight into researchers’ drivers and inhibitors for both sharing and using open research data. For example, Piwowar, Day [8], along with Piwowar and Vision [9] have found that researchers might be driven to share their data openly as this could result in greater visibility of the researcher and thus lead to a greater citation rate. Moreover, researchers might want their study results to be both transparent and verifiable [10], or the policy of a journal in which they want to publish in requires them to openly share their data [11]. Researchers may also be reluctant to openly share data due to the fear of possibly not receiving credit [12], losing possible publication opportunities [13–15], facing possible criticism about data quality [16] or due to data sensitivity [17]. Furthermore, previous research has found that researchers may be driven to use open data because this activity saves time and effort, or because the use of open data can accelerate their overall research progress [18]. Yet, researchers might be inhibited to use open research data due to possible fragmented data and that it is difficult to assess their quality [19, 20] or due to the difficulty finding or accessing reusable data, the difficulty of integrating data and possible data misinterpretation [17].

Despite various emerging data sharing initiatives in the past few decades [21], most raw datasets have still not been openly shared [5]. Prior research has pointed out that the current rewarding system does not sufficiently encourage individual researchers to accomplish open science principles’ best practices such as those involving transparency, reproducibility, openness, and data reuse [22]. In addition, previous research has not had a comprehensive thematic analysis that both explains and integrates the drivers plus inhibitors for both sharing and using open research data. Per Hossain, Dwivedi [23], existing literature has both discretely explored and provided results based on several antecedents to open data adoption (i.e. community participation). Yet, such results might be scattered and a comprehensive overview of factors has not yet been developed. Many studies have addressed both the drivers and inhibitors for sharing and using open research data. Yet, such studies only reveal a rather small part of the full picture. By investigating both data sharing and use, along with individual drivers and organizational contexts and arrangements–all of these create a more holistic understanding of both open research data sharing and reuse.

To fill the existing literature gap, this study’s purpose is to systematically review the literature on both individual researchers’ drivers and inhibitors for both sharing and using open research data. This study defines open research data as structured plus machine-readable data that can be actively published or shared on the Internet, and that ideally also reflects the FAIR principles: Findable, Accessible, Interoperable, Reusable [24, 25]. Open research data can be raw, can be derived from primary data for subsequent analysis or interpretation, or simply can be derived from existing sources held by others [26]. Likewise, both data derived from qualitative and quantitative research altogether are within this study’s scope.

In the subsequent section, this study explains our approach towards the Systematic Literature Review. Thus, this study’s obtained results include both a descriptive analysis and principle themes rooted from the aforesaid literature. Lastly, this study discusses such findings’ implications for future research and practice in which conclusions are further derived from.

## Research approach: Systematic literature review

A literature review reflects “the selection of available documents (both published and unpublished) on the topic that altogether contain information, ideas, data and evidence written from a particular standpoint to fulfil certain aims or express certain views on the nature of the topic and how it is to be investigated, and the effective evaluation of these documents in relation to the research being proposed” [27]. One of the systematic literature review approach’s main advantages lies in its rigor and the applied processes’ overall transparency [28]. Literature reviews have been proven to be useful in various diverse research disciplines such as those of software engineering [29], evidence-based medicine [30], social networks [31], and supply-chain management [32]. In the context of open research data, Fecher, Friesike [11] also found that the systematic literature review approach can be a useful way to “systematically retrieve research papers from literature databases and analyze them according to a pre-defined research question” [p. 3].

Despite the aforesaid advantages of literature reviews, one should also be aware that systematic reviews’ validity might be reduced due to possible ‘publication bias’. This is because publication bias occurs when researchers both selectively report and publish statistically significant positive results of experiments, rather than negative or null results [33]. With this in mind, this study is scoped towards a specific selection of open research data academic articles, along with excludes grey literature, news articles, blog posts and preprints. Literature reviews can be used for various purposes, such as those involving positioning research relative to existing knowledge and building on this knowledge, gaining useful insights on the research topic, introducing relevant terminology and defining key terms, obtaining useful insights on the research methods other scholars have used to study the research topic, along with relating research results to those of others [34]. In this study, a literature review was applied for three reasons. For the first reason, it is done so to both position the identified research relative to existing knowledge and to build on this knowledge. Thus, the following questions were formulated:

a) In which contexts has both open research data sharing and use been investigated by previous research (e.g., research disciplines, countries, types of institutions)?
b) What are both the objectives and contributions of previous research about both open research data sharing and use?
c) What theories and theoretical models have been indicated (e.g., applied, developed, used, tested) in studies about both open research data sharing and use?

For the second reason, it is to gain useful insights in the research methods other scholars have applied to study the research topic. Thus, rendered was the following question:

d) What research designs have been applied in previous research about both open research data sharing and use?

For the third reason, it is to obtain useful insights on this research’s topic–namely regarding the researchers’ drivers and inhibitors for both sharing and using open research data. Thus, rendered were the following questions:

e) What factors drive researchers to openly share their research data with others?
f) What factors inhibit researchers from openly sharing their research data with others?
g) What factors drive researchers to use openly available research data from other researchers?
h) What factors inhibit researchers from using openly available research data from other researchers?

In this study, the Systematic Literature Review approach was applied per Kitchenham [35]. This approach involves five respective steps: (1) identification of studies; (2) study selection; (3) study quality assessment; (4) data extraction; (5) data synthesis. The following paragraphs detail such steps. This study’s Systematic Literature Review approach’s first two steps concern both the research articles’ identification and relevant studies’ selection. Determined was the study selection criteria and selection process, then discoursed were the inclusion decisions. To identify as many relevant articles as possible, a diverse number of databases were searched, namely: Web of Science, ACM Digital Library, and Scopus (includes Elsevier/ ScienceDirect, Springer, Taylor & Francis, Wiley Blackwell, IEEE, Sage, Emerald, Cambridge University Press). For each database, the first 50 results were scanned–sorted by relevance–by carefully reading such results’ respective abstracts and titles. Also searched were three prominent journals in the library and information sciences-related discipline, namely articles involving data sharing research. These three journals were the: (1) “International Journal of Digital Curation”; (2) “Journal of the Association for Information Science and Technology”; (3) “Electronic Library”.

Table 1 lists the search terms applied in this study. Such terms’ selections were not limited to a certain disciplinary or geographical or area, because this would yield a large number of studies with too narrow of a scope. Instead, included were articles pertaining to both research data sharing and use worldwide, coupled with articles from all research disciplines types. Studies were identified in the summer of 2020 and studies published post-December 2019 were excluded. To ensure that this study’s literature review includes more up-to-date information, this study’s paper inclusion period was limited to the last 16 years and thus excluded were papers published before. Ultimately, 101 articles were identified.

**Table 1:** Search terms used in our systematic literature review.

*Construct*	Combinations of search terms used in the systematic literature review
*Motivation*	(data OR “open data”) AND (motiv* OR demotiv*)
*Sharing data openly*	(data OR “open data”) AND (shar* OR provid* OR publish OR releas*)
*Re-using open data*	(data OR “open data”) AND (use OR reuse)
*Influencing factors*	(data OR “open data”) AND (factor OR influence)

As recommended by Jalali and Wohlin [36], the pool of studied articles in the systematic literature review was expanded and complemented using a snowballing technique. Thus, 35 additional relevant articles were identified via the reference lists of the publications that had already been found using search strings—thus enriching the overall literature base. By combining the systematic literature review with the snowballing approach and removing the duplicates, 119 studies were identified that detail research about both open data sharing and use. Applied were both Endnote as a bibliography management tool and Excel Spreadsheets for general search plus search results’ documentation. The raw data from this study’s analyses are available via the 4TU. Centre for Research Data: https://doi.org/10.4121/12820631.v1.

For each of the 119 identified records, their respective abstracts and titles were examined. In this step, 69 studies were excluded due to per below:

- Many studies focused on open government data or open data for businesses (n = 45). As this study is focused on both researchers’ data sharing and use, not considered were factors that impact business or governmental-related open data sharing and use.
- Several studies were excluded as they were considered to be irrelevant to this study’s research question (n = 21), such as studies focused on motivations related to e-commerce or open source. Relevance was determined per how the identified article fits within this study’s aims. This is so to develop a more comprehensive overview of factors that explain why researchers are motivated to openly share and use research data or not.
- Two of the identified records appeared to be workshop descriptions. These appeared in our search as they were published as conference proceedings. As these records did not detail research, they were removed from our sample.
- One record was excluded as it was not accessible.

After this step, 50 studies remained.

A systematic literature review’s third step is to assess the studies’ quality [35]. Especially in the appraisal of qualitative research, this study concurs with Estabrooks, Field [37] that papers of weaker quality should be excluded from systematic literature reviews. Yet, what determines qualitative research quality has been highly prone to both heated debate and criticism [28]. Namely in qualitative research’s systematic review, the study quality’s assessment continues to be a challenge and it might lead to different quality assessments by assessors [28]. Although this challenge cannot be removed completely, this study undertook various measures to reduce bias resulting from it as much as possible. For example, by providing transparency about this study’s assessment procedure and by openly sharing the research data underlying our analysis and findings—thus other scholars were enabled to both cross-check our findings and examine if other interpretations might be possible.

Batini et al. [38] detailed that the four criteria most vital to most literature involving data quality assessment are: accuracy, completeness, consistency, and timeliness. In this study’s systematic literature review, each study was respectively assessed against such aforesaid dimensions. In a detailed manner, such assessments defined the quality assessment criteria using insights from the systematic literature review protocol developed by Bano and Zowghi [39]. This resulted in the creation of the first version of this study’s rubric. When this study started with the quality assessment using this rubric, all of this study’s three authors independently assessed the first six papers. Next, discussed were such assessments’ outcomes that include minor differences in the quality assessment criteria’s interpretation. With this rubric’s further improvements, the final resulting rubric was applied to assess the studies’ quality (see Table 2). Upon this, the remaining studies included in such sample was divided into two. The first half was altogether assessed by this study’s first and second authors. The second half was altogether assessed by this study’s first and third authors. Thus, each article was independently assessed by at least two assessors. All assessors hold both extensive open data field experiences and trainings in qualitative research assessment. No conflicting assessments were found in the assessment’s second round.

**Table 2:** Rubric used for quality assessment in our systematic literature review.

*Quality dimensions (derived from [38])*	Explanation (adopted from [39])
*Timeliness—the study needs to be based on studies published relatively recently (i*.*e*. *in the past fifteen years)*	The study was published in the period September 2004 –December 2019 inclusively.
*Accuracy–the study and particularly the study’s research approach needs to be accurate*	The objectives of the study are clearly stated and the data collection methods are adequately described. Important statements in the paper are supported by references.
*Consistency–different elements of the study need to be consistent*	The study’s design is appropriate with respect to the research objectives and the study’s research questions are answered.
*Completeness–the study’s research approach needs to be described in sufficient detail*	For case studies: the case study context is defined and a clear chain of evidence is established from observations to conclusion.
	For surveys: the authors justify the sampling approach and sample size, population representation, and generalizability are discussed.
	For experiments: variables applied in the study are adequately measured and information about the treatment and control condition is described.

From the 50 identified studies, eighteen studies were removed due to:

- Nine studies did not have clear research questions and/or did not describe the collection of empirical data. Instead, such studies included essays, opinion articles, conceptual studies or studies in which a proposed method, prototype or architecture were detailed.
- Seven articles provided insufficient information for quality assessment. Quality is defined per Kitchenham [35]: an article’s quality is based on the credibility of how a study is both analyzed and conducted, followed by the findings’ importance. And some studies were not subject to peer review, but to editorial review only. These were ultimately left out.
- One study concerned a combined quantitative and qualitative analysis of the eleven responses provided to a questionnaire. The limited number of responses does not allow for quantitative analysis in the form applied by the authors. In addition, the study population was not explained.
- One study appeared to be a shorter version of an extended paper already included in the selection.

**Fig 1:**
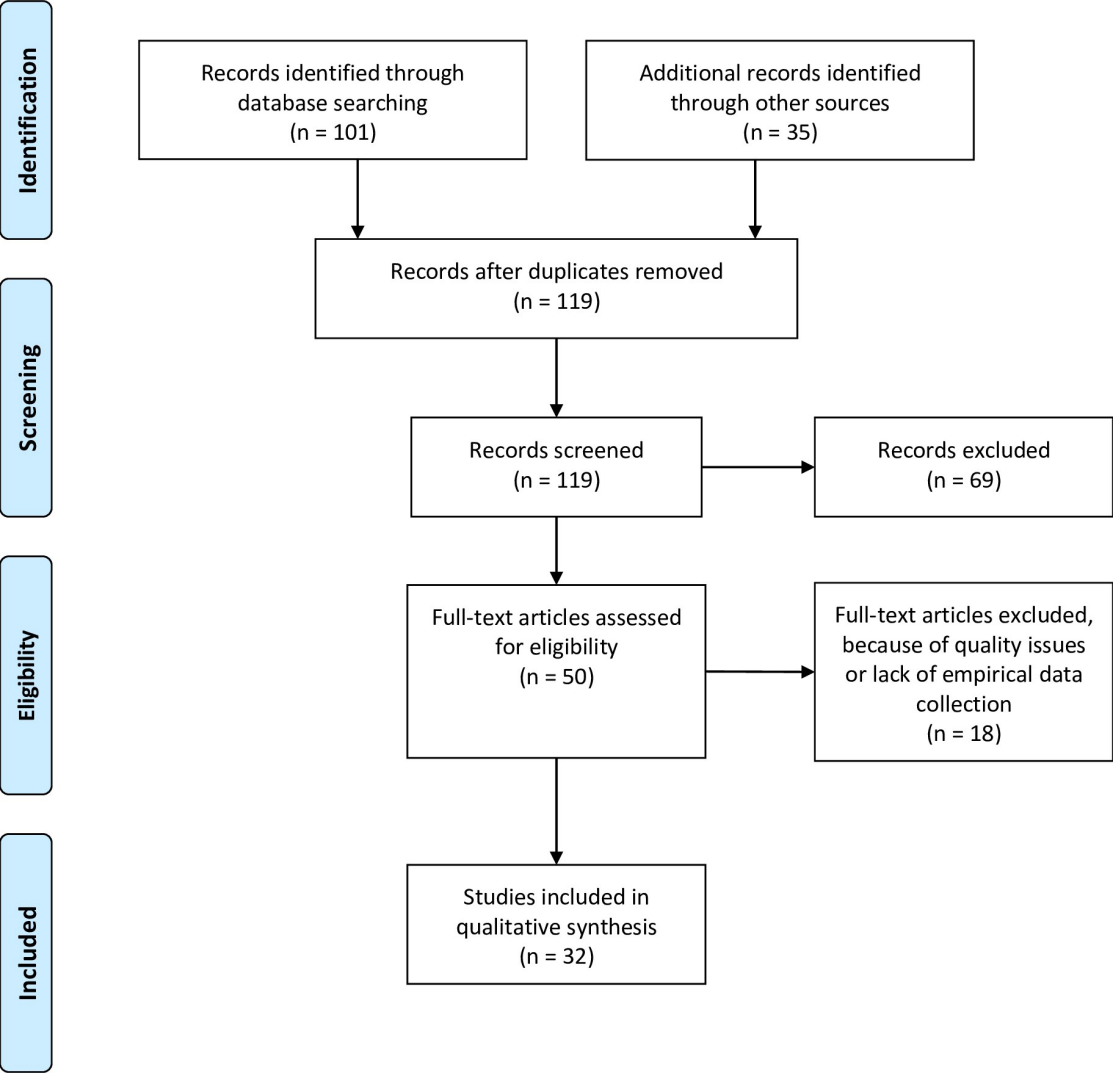
Study selection and assessment (using the PRISMA flow diagram).

The aforesaid steps resulted in a final selection of 32 articles concerned with both drivers and inhibitors for both sharing and using open research data (Fig 1).

In the systematic literature review’s fourth step (the data extraction step), a spreadsheet was applied to keep track of the metadata for each of the selected studies. Table 3 depicts the metadata that were collected for the 32 selected studies that include: general information, context-related information, research design-related information, content-related information, along with information concerning both drivers and inhibitors for both sharing and using open research data. In this study’s final step, information that was obtained via the aforesaid research approach was synthesized. This synthesis’ findings are detailed in the study’s subsequent section.

**Table 3:** Overview of information collected about each of the selected articles.

	*Metadata*	Description
*Descriptive information*	Article number (#)	A study number, corresponding to the study number in the appendix (S1 Table).
	Complete reference	The complete source information to refer to the study, including the author(s) of the article, the year in which it was published, the article’s title and other source information.
	Year of publication	The year in which the study was published.
	Journal / book	The journal or book in which the study was published.
	Website	A link to the website where the study can be found.
	Search terms which led to this article	The search terms (see Table 1) used to identify this article.
	Found through (database)	The database used to find the article.
*Context-related information*	Unit of analysis	The unit of analysis of the selected study in terms of the country, organization, or other specific unit that has been analyzed.
	Research discipline	The research discipline in which data sharing and/or use is investigated (as stated by the authors).
	Period under investigation	Period (or moment) in which the study was conducted (e.g. January 2015-March 2016).
*Research design-related information*	Research method(s)	The methods used to collect data in the selected study.
	Qualitative / quantitative / mixed methods	If the study uses a qualitative, quantitative or mixed methods approach.
	Availability of the underlying research data	If the paper contains a reference to the public availability of the underlying research data (or explains why this data is not openly shared).
	Literature review approach explained?	If the literature referred to in the selected study was systematic or not.
	Quality concerns	If there are any quality concerns (e.g. limited information about the research methods used).
*Content-related information*	Study objective	The study’s objective, as stated by the author(s). If the author(s) did not explicitly state the objective, we derived it ourselves.
	Study’s contributions	The study’s contributions, as stated by the author(s). If the author(s) did not explicitly state the contributions, we derived it ourselves
	Theory mentioned	Does the study mention any theory? If yes, what theory?
	Use of theory	If any theory is mentioned, how is theory used in the study? (E.g. mentioned to explain a certain phenomenon, used as a framework for analysis, tested theory, theory mentioned in the future research section).
*Factor-related information*	Factors driving researchers to share research data openly.	The identified factors positively influencing researchers’ motivations to openly share research data.
	Factors inhibiting researchers to share research data openly.	The identified factors negatively influencing researchers’ motivations to openly share research data.
	Factors driving researchers to use open research data.	The identified factors positively influencing researchers’ motivations to use open research data.
	Factors inhibiting researchers to use open research data.	The identified factors negatively influencing researchers’ motivations to use open research data.
	Does it concern research data opening, research data use or both?	If the study is focused on research data opening, research data use or both.

## Results: Data extraction and data synthesis

Per Kitchenham [35], in this section, the results of the synthesis from the studies collected via the literature review are reported. Extensive descriptive analyses and content analysis were carried out, that are common in information systems-related research [23]. This is to summarize the article attributes and further report the descriptive results. Before the content analysis, several preparatory phases were undergone: metadata extraction, context analysis, and quality analysis (see following sections). Upon accessing all the sampled articles (n = 32), the three assessors first identified and captured metadata plus descriptive information from each article that include both the publication type and year. All the metadata attributes and the described information were altogether collected, cleaned, and organized in a spreadsheet style dataset.

With the descriptive data, the S1 Table (‘Overview of studies included in our literature review’) provides an overview of the 32 studies that detail research into open data sharing and use that this study selected to thus develop the comprehensive factor overview. This appendix too details these studies’ respective objectives. The majority have been published from the years 2010 to 2019 inclusively, except for one article published in 2004 and one in 2007. Most studies (n = 30) have been published in journals, such as the: “PLOS ONE” (n = 7), “Data Science Journal” (n = 3), and “International Journal of Information Management” (n = 3). One dissertation was also included.

Given that the descriptive information was insufficient to cover all the necessary attributes that this study requires, both the context-related information and information about the design of the examined studies was collected, such as the discipline that the article addressed and the period under investigation (context-related), coupled with the possible research approach and possible quality concerns (research design-related). Such forms of analysis were then followed by the content analysis that includes the factors that impact both open research data sharing and use. To reduce the risk of bias in collecting the data, specified were how many studies report each particular factor in the synthesis and made available was the raw underlying research data so that the findings could be further examined. The data underlying this section can be further found here: https://doi.org/10.4121/12820631.v1. In the following sections, this study reports the findings involving the context analysis, research design’s analysis, and content analysis.

### Context analysis

Out of the 32 studies, nearly half of them both examined data sharing and use in the global context or multiple countries (n = 13), namely those involving the United States in tandem with several European countries. Some other studies focus on the United States as the primary nation under investigation (n = 9). Eight studies focus on both open data sharing and use in individual nations such as: the Netherlands, Argentina, Brazil, or the United Kingdom. Whereas, one study focused on both Kenya and South Africa. Twenty-four studies specified the period in which they were conducted, while eight studies do not.

About the research disciplines under investigation, the majority of the articles (n = 25) focused on specific research disciplines such as: biodiversity, sociology, microarray science, psychology, health sciences, earth and space science, genetic and genomic sciences. Eight articles include multiple research disciplines, such as those from the social sciences, humanities, natural sciences, information sciences, engineering, biology, education, law, and business. Two articles did not specify the research discipline(s) under investigation at that time.

### Analysis of research design

As aforesaid in Table 3, the analysis of the research design considered the: (1) research methods (e.g. quantitative) and approaches (e.g. survey); (2) underlying research data’s availability; (3) literature review approach’s transparency; (4) overall quality concerns. In this study’s sample, the division of qualitative and quantitative studies was nearly equal in which fifteen of the 32 selected studies being exclusively quantitative and twelve being qualitative. Five studies applied a mixed-methods approach that combined both qualitative and quantitative research approaches. Fifteen of the 32 studies applied questionnaires as the primary data collection approach. Other research methods often used in open data research were interviews (n = 8) and case studies (n = 5). Thirteen studies applied other data collection approaches such as: quasi-experiments, expert panels, observations, dataset analysis, desktop research, and an analysis of the published papers’ respective number of citations (i.e. scientometric approach).

For nearly half of the studies, it is either unclear if the underlying research data are openly available or the data are not shared openly, since there is no reference to the data’s availability (n = 14). At times, there are references to similar cases in other publications or to reports that use the same research approach, without specifying where the raw research data can be found. Note that a lack of information about where the underlying research data can be found does not necessarily mean that this data is not openly available, as it may have been shared openly without being mentioned in the study itself. This circumstance can happen when the data is only shared after the publication of the article. And in some studies, it is mentioned that all the data was already included in the publication, but in those cases, the data was not shared in a machine-readable format. Sixteen studies do specify where the underlying research data can be found. Of the selected studies, the underlying research data is shared openly via, for example, Dryad, Github, Mendeley Data and an institutional data repository. Some of the shared data is not in a machine-readable format. In two studies, it is mentioned that it is not possible to openly share the underlying research data due to possible confidentiality issues.

As a final topic involving research design, we examined if there were any overall quality concerns about the 32 analyzed studies’ quality. For four articles, there are at least some concerns. For example, in one study, the investigated cases had been described and analyzed, while the case study selection criteria had not been specified. As another example, in one study it was unclear how many case studies have been conducted and exactly what they were about, as there was only a reference to an OECD report that contains this information. In another study, some information about the information sources of the case studies that were carried out was missing.

### Content analysis

The majority of the investigated studies (n = 18) did not mention any theory (this study had a narrow view on what comprises theory), while fourteen studies mention one or more theories. Seven out of these fourteen mention the “Theory of Planned Behavior” (TPB), two mention “Institutional Theory”, two mention “Technology Adoption Model” (TAM), and two mention an integrated theory of the “Unified Theory of Acceptance and Use of Technology” (UTAUT), along with the two-stage “Expectation Confirmation Theory of Information Systems” (IS) continuance (ECT). Other theories were mentioned only by one study, namely the: “Theory of Reasoned Action” (TRA), Organizational theories (commons-based peer production, wisdom of the crowds and collective intelligence), “Unified Theory of Acceptance and Use of Technology”, “Grounded Theory”, “Motivation Theories” (e.g. Expectancy Theory, Reinforcement Theory, The Multi-Motive Information Systems Continuance Model), and “Coordination theory”.

The fourteen studies that mention theory applied it in various ways. Eleven studies applied theory to develop the theoretical research framework or model and/or to test hypotheses. The authors of these studies reflect on the theory in relation to their research model. One of those eleven developed a theory as the research outcome, while building on existing theories. One study mentioned the theory in the discussion section and examines the implications of the study on existing theories, without using the theory in other parts of the research. One study only mentions the theory in the recommendations for future research without using it elsewhere (Table 4). The discussion section further explores the potential and opportunities for using theories in open research data studies.

**Table 4:** Overview of theories and the way they are used in the selected studies.

*The way theory is used in open research data studies*	Name of selected theory	Source
*Applied theory (e*.*g*. *to develop the theoretical research framework / model*, *to test hypotheses and to reflect upon)*	Theory of Reasoned Action (TRA)	Curty, Crowston [40]
	Theory of Planned Behavior (TPB)	Harper and Kim [41], Joo, Kim [17], Kim and Adler [42], Kim and Yoon [43], Yoon and Kim [44], Zenk-Möltgen, Akdeniz [45]
	Technology Adoption Model (TAM)	Yoon and Kim [44]
	Integrated Unified Theory of Acceptance and Use of Technology (UTAUT) with the two-stage expectation confirmation theory of Information Systems (IS) continuance	Zuiderwijk [19], Zuiderwijk and Cligge [46]
	Institutional theory	Kim and Adler [42], Kim and Yoon [43]
	Coordination Theory	Zuiderwijk [19]
	Grounded Theory	da Costa and Leite [47]
	Motivation theories (e.g. Expectancy Theory, Reinforcement Theory, The Multi-Motive Information Systems Continuance Model)	Zuiderwijk and Spiers [48]
*Discussed the findings of the research in relation to the study*	Organizational theories: commons-based peer production, wisdom of the crowds and collective intelligence	Fecher, Friesike [11]
*Mentioned theory (in the recommendations for future research)*	Theory of Planned Behavior (TPB)	Sayogo and Pardo [49]
	Technology Adoption Model (TAM)	Sayogo and Pardo [49]
	Unified Theory of Acceptance and Use of Technology (UTAUT)	Sayogo and Pardo [49]
*Developed theory (as an outcome of the study)*	Design theory for open government data infrastructures	Zuiderwijk [19]

### Analysis of factors influencing open research data sharing and use

#### The focus on open research data sharing, use or both

For the 32 studies analyzed, it was examined how many of them mentioned: (1) researchers’ drivers for sharing research data openly; (2) researchers’ inhibitors for sharing research data openly; (3) researchers’ drivers for using open research data, (4) researchers’ inhibitors for using open research data (see Tables 5 and S2–S5).

**Table 5:** Overview of the studies included in our systematic literature review.

No.	Authors	Title	Sharing data openly		Open data use	
			Drivers	Inhibi-tors	Drivers	Inhibi-tors
*1*	Arza and Fressoli [4]	Systematizing benefits of open science practices	X		X	X
*2*	Arzberger, Schroeder [50]	Promoting access to public research data for scientific, economic, and social development	X	X	X	X
*3*	Bezuidenhout [51]	Technology Transfer and True Transformation: Implications for Open Data		X		X
*4*	Campbell [2]	Access to scientific data in the 21st century: Rationale and illustrative usage rights review	X	X	X	X
*5*	da Costa and Leite [47]	Factors influencing research data communication on Zika virus: a grounded theory	X	X	X	X
*6*	Cragin, Palmer [52]	Data sharing, small science and institutional repositories	X	X		
*7*	Curty, Crowston [40]	Attitudes and norms affecting scientists’ data reuse	X		X	X
*8*	Enke, Thessen [10]	The user's view on biodiversity data sharing—Investigating facts of acceptance and requirements to realize a sustainable use of research data	X	X	X	X
*9*	Fecher, Friesike [11]	What drives academic data sharing?	X	X	X	
*10*	Ganzevoort, van den Born [53]	Sharing biodiversity data: citizen scientists’ concerns and motivations	X	X		
*11*	Grechkin, Poon [6]	Wide-Open: Accelerating public data release by automating detection of overdue datasets	X	X	X	
*12*	Haeusermann, Greshake [18]	Open sharing of genomic data: Who does it and why?	X	X	X	X
*13*	Harper and Kim [41]	Attitudinal, normative, and resource factors affecting psychologists’ intentions to adopt an open data badge: An empirical analysis	X	X		
*14*	Joo, Kim [17]	An exploratory study of health scientists’ data reuse behaviors: Examining attitudinal, social, and resource factors	X	X	X	X
*15*	Kim and Adler [42]	Social scientists’ data sharing behaviors: Investigating the roles of individual motivations, institutional pressures, and data repositories	X	X		
*16*	Kim and Yoon [43]	Scientists’ Data Reuse Behaviors: A Multi-Level Analysis			X	X
*17*	Mooney and Newton [13]	The anatomy of a data citation: Discovery, reuse, and credit	X	X	X	X
*18*	Piwowar and Vision [9]	Data reuse and the open data citation advantage	X	X	X	
*19*	Piwowar, Day [8]	Sharing detailed research data is associated with increased citation rate	X	X	X	
*20*	Raffaghelli and Manca [54]	Is there a social life in open data? The case of open data practices in educational technology research		X	X	X
*21*	Sá and Grieco [1]	Open data for science, policy, and the public good	X	X		
*22*	Sayogo and Pardo [49]	Exploring the determinants of scientific data sharing: Understanding the motivation to publish research data	X	X	X	
*23*	Schmidt, Gemeinholzer [55]	Open Data in Global Environmental Research: The Belmont Forum’s Open Data Survey	X	X		X
*24*	Tenopir, Allard [56]	Data Sharing by Scientists: Practices and Perceptions	X	X	X	
*25*	Wallis, Rolando [57]	If we share data, will anyone use them? Data sharing and reuse in the long tail of science and technology	X	X	X	X
*26*	Yoon [58]	Data reusers' trust development			X	X
*27*	Yoon and Kim [44]	Social scientists’ data reuse behaviors: Exploring the roles of attitudinal beliefs, attitudes, norms, and data repositories			X	X
*28*	Zenk-Möltgen, Akdeniz [45]	Factors influencing the data sharing behavior of researchers in sociology and political science	X	X		
*29*	Zimmerman [59]	Not by metadata alone: The use of diverse forms of knowledge to locate data for reuse	X	X	X	X
*30*	Zuiderwijk [19]	Open data infrastructures: The design of an infrastructure to enhance the coordination of open data use	X	X	X	X
*31*	Zuiderwijk and Cligge [46]	The acceptance and use of open data infrastructures-drawing upon UTAUT and ECT			X	X
*32*	Zuiderwijk and Spiers [48]	Sharing and re-using open data: A case study of motivations in astrophysics	X	X	X	X

‘X’ means that at least one factor in the particular category was mentioned in the study.

Of the 32 records studied, six of them focused exclusively on data sharing and do not mention any factors related to the motivation to use open research data. Four studies focused exclusively on open research data use and do not mention factors related to open research data sharing. Twenty-six articles mention factors related to both open data sharing which can be explained by the interdependence between these two activities: data users depend on data providers in order to get research data, while data providers make research data available to data users and depend on them for feedback, development of the field of research and possible future collaborations. However, despite a few exceptions [e.g., 17, 19, 40, 50], the focus of the majority of the studies addressing both data sharing and use is on research data sharing. These studies only briefly mention factors related to open data use, as it is not their main topic. Our study confirms research by Joo, Kim [17] in the sense that “a relatively smaller body of research has focused on data reuse as compared to data sharing” (p. 390).

#### Principal themes

For each of the 32 analyzed articles, the factors that may drive or inhibit researchers to openly share their research data with others were identified, along with the factors that may drive or inhibit researchers to use open research data shared by others. The S2–S5 Tables provide this analysis’ detailed results. It was found that various articles refer to similar constructs. Also, this study categorized the constructs of the influencing factors into the following eleven categories:

1. *The researcher’s background*. This category concerns factors related to the researcher’s personal characteristics and research background that might impact one’s open data sharing and use behavior altogether.
2. *Requirements and formal obligations*. This refers to whether formal obligations are in place, such as those imposed by the project’s funder and if other forms of requirements are experienced, such as (in)formal policies.
3. *Personal drivers and intrinsic motivations*. This refers to intrinsic motivations for both open research data sharing and use.
4. *Facilitating conditions*. This refers to anything that can facilitate open research data sharing or use.
5. *Trust*. This refers to how the level of trust a researcher has influences their open research data sharing and use behavior altogether.
6. *Expected performance*. This concerns factors that may influence the performance of researchers who share and use open research data or not.
7. *Social influence and affiliation*. This concerns factors related to social influence and affiliation that impact if a researcher is driven to both share and use open research data.
8. *Effort*. This refers to the effort needed for a researcher to openly share or use research data.
9. *The researchers’ experience and skills*. This refers to previous experience that a researcher has with open research data sharing and use and skills required for this activity, coupled with how this impacts future research data sharing and use altogether.
10. *Legislation and regulation*. This concerns the impact of factors related to legislation and regulation on research data sharing and use behavior altogether.
11. *Data characteristics*. This refers to the influence of data characteristics on if a researcher both shares and uses open research data.

In the following sections, the factors that drive and inhibit researchers to openly share their research data with others are discussed, along with the factors that drive and inhibit researchers to use open research data shared by others. The factors are discussed with the aforesaid categories.

#### Factors driving and inhibiting researchers to openly share their research data

This section answers the question: ‘What factors *drive* researchers to openly share their research data with others?’ and ‘What factors *inhibit* researchers from openly sharing their research data with others?’ Table 6 depicts both such drivers and inhibitors. It shows that several factors relate to different sides of the same coin. For example, the factor ‘level of involvement in research activities’ refers to the finding that individuals who work solely in research, in contrast to researchers who have time-consuming teaching obligations, are in fact more likely to make their data available to other researchers [11]. Thus, for researchers who solely work in research, the ability to focus on research without having to teach can be considered a driving factor, whereas for researchers who have time-consuming teaching obligations, this can in fact be considered an inhibiting factor. Other factors are more specifically related to either drivers for open data sharing, such as the increased pressures to release data [57], or to inhibitors for data sharing, such as the time and effort it takes to openly share research data [11].

**Table 6:** Thematic analysis of researchers’ drivers and inhibitors for sharing research data openly, identified in the selected 32 studies.

*Themes*	Drivers for researchers to share their research data openly	Inhibitors for researchers to share their research data openly
*The researchers’ background*	Disciplinary practice [11, 56] and culture of data sharing [40]	Level of involvement in research activities (individuals who work solely in research, in contrast to researchers who have time-consuming teaching obligations, are more likely to make their data available to other researchers) [11]
	Research discipline/area (e.g. Biology researchers are more inclined to openly share data than Medicine and Pharmacy [47] and more data sharing in political science than in sociology) [45]	Seniority in the academic system (non-tenured researchers are less likely to share their research data openly) [11]
	Culture: organizational culture [11], open-working academic culture [47], a supportive data sharing culture [48]	Gender: the probability of not publishing data sets is higher than the probability of publishing some, most, or all of the datasets for male respondents [49]
	Level of involvement in research activities (individuals who work solely in research, in contrast to researchers who have time-consuming teaching obligations, are more likely to make their data available to other researchers) [11]	Nationality in relation to national research policies (e.g. German and Canadian scientists are more reluctant to share research data publicly than their US colleagues) [11]
	Seniority in the academic system (non-tenured researchers are less likely to share their research data openly) [11]	Researchers’ age: younger researchers (age 20–35) are more concerned about the impact of data release compared to older researchers (age 51 and older) [55]
	Researcher’ age [56], where younger researchers are more inclined to openly share their data [47]	
	Gender: the probability of not publishing datasets is higher than the probability of publishing some, most, or all of the datasets for male respondents [49]	
	Country and geographic location [17, 56]	
*Requirements and formal obligations*	Increased pressure to release data [57]	Study sponsors, particularly from industry, may not agree to release raw detailed information [8]
	Compliance with governmental directives [40]	Losing funding opportunities [13]
	Mandates for data management plans from federal agencies [56]	Lack of funder requirements to publish data [55]
	Mandates for research data sharing [40], e.g. data sharing requirements and pressures by journals [41, 42, 47, 55]	Too many data policies apply [55]
	Received funding from government agencies [11, 42]	
	Funder’s policies [55]	
	University policies requiring data release [48]	
	Financial compensation [11]	
	Ethic codes [41]	
	Exterior public data is shared automatically [48]	
*Personal drivers and intrinsic motivations*	Character traits (Big Five: openness to experience, conscientiousness, extraversion, agreeableness, neuroticism) [11]	Character traits (Big Five: openness to experience, conscientiousness, extraversion, agreeableness, neuroticism) [11]
	Individual incentives [17], e.g. wanting to learn about yourself [18]	Commercialization of research findings [11]
	Perceived behavioral autonomy and self-efficacy to be able to share data [45]	Fear of few reuse [51]
	Positive attitude towards data sharing [45]	Laziness [55]
	Personal drivers [48]	Negative attitude towards data sharing [45]
	Sense of responsibility about dissemination and recognition of research results [55]	
	Personal commitment to open data and respond requests from data users [55]	
	Better inform society and foster new processes of learning [4]	
	Equal access to publicly funded data [4]	
	As data was generated with public money it should be made public [10]	
	Reuse value for many years [52]	
	Allowing access of the data for more disciplines and for researchers from different backgrounds [42]	
	Offer individuals the opportunity to better understand the social and physical world in which we all live [50]	
	Provide decision-makers with the necessary facts to address complex, often trans-national problems [50]	
	Encourage validation and verification of research results [2, 56] and enable falsification [11]	
	Help to identify errors and discourage research fraud [8, 9]	
	Permitting in-depth public scrutiny by making it easier to analyze, process and combine data [19]	
	Encourage multiple perspectives [8, 42] and allow other researchers to explore new interpretations of data [17, 56], ask new questions [57] and test different hypotheses [42]	
	Allow valuable resources to contribute far beyond their original analysis [9]	
	Facilitating other researchers' ability to pursue new lines of research [57]	
	Facilitating comparisons between methods and sites [57]	
	Data reuse can lead to more findings from the same dataset and increase the knowledge in the field [17]	
	Personal drivers / intrinsic motivations: better science, move the field forward more quickly and easily [48]	
	Sense of responsibility about acceleration of scientific research [55]	
	Usability [48]	
	Possibility to better advance the area of research [52]	
	Size of the research community and the extent to which data is viewed as a tremendous asset [52]	
	Encourage economic development, spur innovation [2]	
	Identify synergies [11]	
	Accelerated scientific progress [11, 17, 57] and contributing to the advancement of research [18, 42]	
	Gaining new insight for data-driven research [19]	
	Enable citizen science and encourage public activism [1]	
	Improved discoverability [9, 17]	
	Extending research from prior results [56]	
	A focus on best work through data availability [9]	
	Generation of new datasets, information, and knowledge when data from various sources are combined [19]	
	Educating researchers about the consumer side of open data practices [17]	
	The ability to review works derived from the dataset [56]	
	Lack of concerns about ethics and commercial potential of data [48]	
*Facilitating conditions*	ICT facilitation (internet hosts per person, percentage of computers per household, continued rate of growth of chip, storage, and network technology capacity) [50]	Financial arrangements (and budgets) [50] and financial resources [11, 41]
	Infrastructure [17, 57], appropriately designed technological infrastructure [50]	Financial barriers: loss of potential licensing revenue that would accrue to inventors of patentable discoveries [1]
	Appropriate information systems [47]	Technical challenges [17, 50]
	Richer investment of funding, labors, scale, and infrastructure [57]	Lack of appropriate infrastructure [57]
	Availability of (large) data repositories [13, 17, 41, 42, 47] and archives [13]	Lack of a data repository [42]
	The ability to grow storage and access capabilities and still operate reliably and efficiently [2]	Lack of facilitating platforms [48]
	Continued and dedicated budgetary planning and appropriate financial support [50]	A lack of information systems to disclose raw research data in certain research disciplines (e.g. medicine) [47]
	Adequate funding for the treatment and availability of data [47]	Level of openness of ICT tools which help in opening the data [1]
	Specific funding for the management of research data [47]	Long embargo period, short reuse value [52]
	Consent, e.g. informed consent or contractual consent [11]	Lack of tools to observe data metrics [54]
	Short embargo period [52]	Organizational: institutional members sometimes resist change [1]
		Lack of time [48]; there is not enough time to organize the data [41]
		Structural conflicts and managerial practices in organization (e.g. security reasons, financial interest) [49]
		Communication of the open data results [1]
		Small science (that has less investment funding, labors, scale, and infrastructure) [57]
		Differences in available resources (equipment) which slows down the pace of research. The specter of “being scooped” due to the slower pace of research) [51]
		The helplessness of changing the pace at which data are generated [51]
		Older equipment, poor maintenance and technical support and infrastructural challenges (such as power provision) [51]
		The limited availability of technologies that underpin data engagement activities (e.g. lack of ICTs for reuse, lack of online platforms, lack of appropriate software, lack of analysis procedures, lack of ICTs for curation and storage; lack of analysis software) [51]
*Trust*	Trust [17, 52]	Lack of trust [52]
	Understanding what users may, or may not, do with data in online data repositories [2]	Issues of ethical responsible use of shared data [49]
	Having a say in the data use [11] and the ability to place conditions on data access [56]	Concerns about data integrity [17]
	Data security conditions [11]	Loss of control [10], such as lack of control of the scientific findings and conclusions derived from the data [1]
	Minimal privacy risks [18]	Someone may draw wrong or inappropriate conclusions [10, 52]
	Lending more credibility to research findings [42]	Fear of the misinterpretation of open data [19, 41, 49, 55]
	Facilitated credibility determination [9], since replication and verification is made immediately possible [42]	Fear of misuse of open data [19, 41, 49, 52, 55]
	Reproducibility of results and the fact that anyone can access the data, improve the quality of the research [48]	Data misuse incidents [52]
	Data availability provides safeguards against misconduct related to data fabrication and falsification [56]	Flawed interpretation [11]
	Ensure the validity of the data by multiple users [1]	Potential harm [11]
	Well-managed, long-term preservation helps retain data integrity [56]	Level of knowledge about the data requester [11]
	Transparency of study results [10], research methods and processes [18]	Unclear intent [11]
	Good management of data integrity over time [2]	Difficulty in establishing trust in others' data [57]
	Using measures that make the collection and interpretation of the data easier [59]	"Gift culture" of scholarship (i.e., researchers exchange valuable data through only trusted relationships, not for the public) [57]
		Fear to harm the reputation of the data publisher [19]
		Fear of commercial or competitive misuse [11]
		Supplementary information and laboratory sites are transient [8]
*Expected performance*	Performance [11] and performance expectancy [48]	Reduces expected performance [48]
	Open up opportunities to participate in new international projects widening local scientists’ networks [4]	Fear of loss of data autonomy (e.g., control over unpublished data in publicly accessible online database) [10]
	Network with other scientists for interdisciplinary studies [10]	The desire for personal control of one’s research products [52]
	Potential for collaboration among scholars with similar research interests [41]	Fear of receiving no credit or recognition [13, 50, 55]
	Professional exchange [11]	Lack of proper reward for sharing data [52, 55, 59]
	Effective data preservation and archiving [2]	Someone else publishing with no reward given to the sharer since there is no system of acknowledgement [13]
	Increase scientific efficiency [4]	References to the name of the data creators and publishers are scarce or not prominently featured (mostly references to the dataset title) [13]
	Through interaction with other actors, research agendas could be better guided towards solving problems affecting a specific group [4]	Improper citation of data [52]
	Finding cheaper solutions to societal problems [4]	Lack of recognition of the citation of the research data as compensation for the effort involved in collecting the data for researchers [47]
	Help local problems to become visible and better communicated [4]	Concern about losing an advantage in their research area [17]
	Other people can offer inputs to develop final solutions [4]	Decrease of their own competitive advantage, whether future publishing opportunities, information trade-in-kind offers with other labs, or potentially profit-making intellectual property [8]
	Appropriate reward structures [13, 50] and recognition for data sharing [11]	Fear of results scooping additional analyses researchers have planned for the future [9]
	Institutional and professional recognition [41]	Fear of data scooping [48, 49], missing out on future publication opportunities [13, 41]
	Being acknowledged [47]	Perceived career risk [42]
	Perceived career benefit [42]	Concerns about protecting the researcher's right to publish their results first [57]
	The possibility of publishing the research results in journals of great international prestige [47]	The desire to publish results before releasing data [55]
	Systematic visibility of the data source [50]	The communication of research data does not receive as much academic prestige as papers [47]
	Increased visibility and relevance of research output [17] [47]	Losing funding opportunities [13]
	Researchers' visibility in the community increases [10, 48]	Losing commercialization opportunities [42]
	Increased visibility for the institution(s) where the research was conducted [47]	Criticism on data or analyses [10]
	Increasing citation rates (of datasets and publications) [8, 9, 40, 41, 48]	Investigators may be afraid that other researchers will find errors in their results [9, 48]
	Increased researcher profile [48]	Fear that the original conclusions may be challenged by a re-analysis, whether due to possible errors in the original study, a misunderstanding or misinterpretation of the data, or simply more refined analysis methods [8]
	Receiving proper data citation credit, formal citation [13]	Fear that additional relationships will be discovered in the data [8]
	High availability of comparable datasets for comprehensive analyses [10]	Scientists' reputation at risk [52]
	The acknowledgement of the dataset's originator in terms of appreciation (e.g. co-authorship on publications, formal acknowledgement of the data providers, opportunity to collaborate with others) [49]	Fear that researchers will be deluged with requests for assistance, or need to spend time reviewing and possibly rebutting future re-analyses [8]
	Collaboration [48]	Incentives and merit system (lack of sufficient rewards and incentives for researchers) [49]
	Demonstrating the value of researchers’ own accomplishments [57]	Incentive systems that favor publishing articles over publishing data [57]
	Generate wealth through the downstream commercialization of outputs [50]	Researchers may lose the ability to barter data privately, thus creating a disincentive for deposit [57]
	Greater returns of public investment in research [50]	Limited data usability [48]
	Improving the predictability of genetic testing [18]	Concerns that if data would be released it would not be reused by international peers because of anxiety linked to the equipment used to produce it [51]
	Review and quality improvement [11]	
	More evaluation capability (e.g. other researchers testing the data and hypotheses [2]	
	Allow researchers to confirm the findings of the original publication or to test different hypotheses [41]	
	Providing evidence to support an analytics framework and decision [42]	
	Promulgating technology as a basis for others' research [57]	
	Professionalism (build on codes of conduct and ethics of the scientific community) [50]	
*Social influence and affiliation*	Social responsiveness [4] and standard social norms [41]	The culture of open sharing (promotion for academe is tied to publication and not data) [49]
	Perceived social pressure to share data with others [45]	
	Code of conduct and related normative standards of professional scientists and their communities [50]	
	Subjective norm [41]	
	Perceived normative pressure [42]	
	Peer pressure to share data [8]	
	Attitudes toward data sharing [17, 42]	
	World-wide attention to the need to share and preserve data [56]	
*Effort*	The expectation that data will be reused [40]	(Perceived) effort [11, 41, 42, 47, 49]
	Avoidance of duplication of work [2, 41, 48, 57]	Required manual efforts [6]
	Increase efficient use of funding and population resources by avoiding duplicate data collection [8, 9]	Individual investment needed to preserve and manage data [57]
	Efficient and optimized use of resources [1, 48, 56]	Time investment (the amount of time they would have to invest to get the data ready to share) [8, 10, 11, 47, 49, 52]
	A source for researchers to consult when considering how to build upon existing studies [42]	Large amount of work [52]
	Saving time involved in data collection [41, 48]	Making data from the long tail discoverable and reusable is emerging as a major challenge [57]
	Reduced research costs [17, 41, 42]	The amount of time or costs that it takes to properly document the data so that it is useful for others [55]
	Increased data use [9]	The data have to be formatted, documented, and released / uploaded [8, 9]
	Tailored data management approaches that meet the needs of researchers [50]	Difficulty of using standards for data sharing [55]
	Institutional models that meet the needs of researchers [50]	No acknowledgement for researchers’ effort [10]
	Organizational support for data management [49]	Technology-related limitation (e.g., reluctance to use online databases because of complex user inter- faces making data entry time consuming) [10]
	Assistance with data management across the data lifecycle [56]	Complicated to release data [8]
	Cleaning, processing, refining and analyzing data already during the research instead of afterwards [52]	Operational: conveying information to the public is not always straightforward [1]
	Technical support [11]	Quality of the open data platforms and credibility [1]
	Software and equipment that reduces the effort required by researchers in producing and disclosing data [47]	Authorship issues and getting permission from all partners in large collaborations [48]
	Repositories reducing the effort required for data registry [47]	Qualitative analytic work [42]
	Identifying the web API for dataset access [6]	
	Adapting the query-result parser to distinguish between invalid UIDs, datasets that have been released, and datasets that remain private [6]	
	To share portions of a dataset rather than to share the whole dataset [59]	
	The researcher has not collected the raw data directly him or herself [48]	
	Quantitative analytic work [42]	
*Researchers’ experience and skills*	Experience with past data sharing [45, 48]	Skills and knowledge (missing knowledge further relates to poor curation and storing skills) [11]
	Data management skills [49]	Lack of expertise [51]
	Knowledge of metadata and its practices [41]	
	Useful for training new researchers [8, 9, 50]	
	Contribute to the education of students [42]	
	Replication studies serve as training tools for new generations of researchers [56]	
	The hiring of data specialists; [47]	
	The possibility of data management consultation [52]	
*Legislation and regulation*	Legislation and regulation [48]	Legal rights and restrictions [2, 19, 49]
	Clear and transparent data policy [53] and data sharing policy [11]	Licensing terms [50]
	Formal organizational policy [56]	Considering licenses a burden [55]
	Policies with data management across the data lifecycle [56]	Concerns about too restrictive licenses (in particular Non Commercial, Share Alike) [55]
	Support from National and local governments (in terms of policies, programs, management practices) [50]	Difficulties in understanding licenses [55]
	Journal policies [11, 42]	Unclear what ‘openness’ means (large variety of licenses) [2]
	National laws and international agreements [50]	Intellectual property right issues [13, 17, 55] and restrictions on use for private intellectual property rights [50]
	Legal and policy requirements (e.g. significance of citation, legal agreement, statement of use, conditions of use, and approval for reuse) [49]	Priority rights for publications [11]
	Regulatory pressure [17]	Fear of potential violation of property rights (intellectual property or patent issues) [10]
		Legal issues [10, 55]
		Concerns about legal liability for data or release of data [55]
		Issues of ownership [11, 50, 59]
		Right of use [11]
		Data sensitivity [17, 19]
		For certain types of data the law prohibits their publication [19]
		Privacy-related concerns [11, 17–19, 41, 42]
		Confidentiality issues [10, 11, 42, 55]
		Contracts with industry sponsors [42]
		Data sources may be copyrighted such that the data subsets cannot be freely shared [8, 11]
		Informed consent agreements may not obviously cover subsequent uses of data and de-identification can be complex [8]
		Legal implications: public access may negatively impact national security [1, 50]
		Datasets created by multiple organizations which have different levels of security, different policies and which have to comply with different laws; all need to give permission for the disclosure of the data [19]
		Privacy and the protection of trade secrets [50]
*Data characteristics*	Data characteristics [48]	Lack of data standards [10, 49, 55]
	Interoperability (and international agreement on interoperability) [11, 50]	Issues of data standards and protection [49]
	Data documentation and metadata, metadata standards [11]	Metadata is not always consistent [57]
	Form of data appropriate for data sharing [52]	Data quality issues [10, 11, 19, 49]
	Data format appropriate for data sharing [52]	Biased data [19]
	Formatting standards [11]	Local contexts and specificity (e.g. the complexity of the data): specificity of purpose, specificity of events, specificity of methodology, and the duration of research [49]
	Easily digestible form [53]	The mobility of data (i.e., data is hard to be moved to other facilities) [57]
	Data management [11]	Data sensitivity (e.g., no distribution to patient data) [47]
	Creating regular expressions for dataset identifiers [6]	Privacy issues [47]
	Effective data quality controls [50]	Data format and form not appropriate for data use [52]
	More data production [50] and data storage [11]	Size of data [48, 55]
	Data security, tools and applications [11]	The large volume of the data [48]
	Data involves no human subjects (e.g., patients) [47]	Dataset too large to share [52]
	Data's nature is quantitative [47]	Data's nature is qualitative [47]

Some factors might fit in multiple categories. For example, one study refers to the inhibiting factors of the “cost of sharing (e.g., time and effort)” [49]. As this factor relates to effort that a researcher needs to put into openly sharing research data, but also to facilitating conditions such as time restrictions. When a factor is related to multiple categories, chosen is the category that we found to be most closely related. For this particular example, we chose the category of effort, as effort was explicitly mentioned by the study’s authors.

Many of the identified drivers for openly sharing research data relate to the ‘personal and intrinsic motivations’, ‘expected performance’ of researchers, and required ‘effort’ involved in openly sharing research data. The identified inhibitors for open data sharing mostly relate to ‘legislation and regulation’, ‘facilitating conditions’ and ‘expected performance’–essentially in the sense that opening up research data can also lead to a worse performance.

#### Factors driving and inhibiting researchers to use open research data from other researchers

This section discusses the factors that drive or inhibit researchers to use openly-available research data from other researchers. Table 7 depicts the inhibitors for researchers to use open research data from other researchers. Similar to research data sharing, several factors can be either drivers or inhibitors, depending on their respective level. For example, both “trust in data producers” [40] and “trust in other researchers’ measurement” [17] are factors that can drive researchers to use open research data, whereas, lower levels of trust and trust concerns [19] can inhibit open research data use. Additionally, for open research data use, we identified several factors that can fit in multiple categories. For instance, the factor “costs associated to training potential data users” [4] could fit both in the category of experience and skills or facilitating conditions. Thus, this factor can be placed in the category of experience and skills as training is strongly related to experience and skills needed for open data use. Yet, this factor would also have fit in the category of facilitating conditions as training might be seen as a condition that facilitates open data use. Drivers for open research data use namely relate to personal and intrinsic motivations, along with the researchers’ expected performances. Likewise, the identified inhibitors for open research data use namely relate to effort and data characteristics altogether.

**Table 7:** Thematic analysis of researchers’ drivers and inhibitors for using open research data, as identified in the 32 selected studies.

*Themes*	Drivers for researchers to use open research data	Inhibitors for researchers to use open research data
*The researchers’ background*	Research / academic discipline [17, 40]	-
	Disciplinary climate (a sense of community and openness to other researchers affiliated in the same field) [17]	
	Research climate [43]	
	Considered data reuse a prevalent research practice in their research communities [44]	
	Traditions [40]	
	Country [17]	
	Sector [17]	
*Requirements and formal obligations*	Policy [17]	Varying policies on access and reuse across countries [2]
	Peer pressure [40]	Ethical bottlenecks [18]
*Personal drivers and intrinsic motivations*	Fun to explore data [18]	Attitude (perceived concern) [17]
	Reinforces open scientific inquiry [50]	Scientists’ attitudes [40]
	Encourages diversity of analysis and opinion [50]	Negative first impressions [58]
	Promotes new research [50]	
	Stimulating economic growth, replication and validation of research [2]	
	Enhancing transparency and reproducibility of the scientific enterprise [40]	
	Scientist’s beliefs and attitudes [40]	
	Feeling worth (e.g., the feeling that the time spent on data reuse is time well spent) [40]	
	Believe data reuse is good [44]	
	Individual willingness [40]	
	Replication of research results [11]	
	Accelerate research [18] and increase the knowledge in the field [17]	
	Advance our understanding of health and disease [18]	
	Value users attach to being tested [18]	
	Explore new interpretations of data [17]	
	Intention to reuse data [17, 43]	
	Data being used enhances public trust and knowledge of the discipline [17]	
	Provides a democratic scientific knowledge sharing platform: "Open access increases the pool of information available to anyone not just scientists" [4]	
*Facilitating conditions*	Facilitating conditions [48]	Lack of facilitating conditions [48]
	Digital tools (e.g. the possibility to involve more actors in data collection through citizen science platforms, not restricted by physical or cognitive distance) [4]	Lack of availability of data [19]
	An open data infrastructure [19], a robust infrastructure for long-term use [50]	Heavy reliance on the methods and techniques data producers employed to obtain, organize and code the data [40]
	The availability of data repositories [17, 43, 44]	Technical bottlenecks [18]
	A large data repository to foster data sharing and reuse culture [17]	Lack of the necessary infrastructure for quick data analysis [47]
	Technical support to ease the process (specialized software or programs) [43]	The lack of approaches that offer both precision and recall when it comes to locating data for reuse [59]
	The possibility to cite and attribute datasets, to foster a scholarly communication system that allows for identification, retrieval, and attribution of research data [13]	Doubts about the long-term availability of the infrastructure [19]
	Organizational environment [17] and institutional support [17, 43] (any possible assistance available that researchers could acquire from their affiliated institutions or organizations, particularly technical or human help) [17]	Lack of interaction support and tools [19]
	Human resource for question (advisors, data reuser groups, data producers) [43]	Search options for open datasets are limited [19]
	Availability of internal resources [43]	Searching for OGD in multiple languages is often not supported [19]
		Lack of support for data analysis [19]
		Interaction related to open data use is limited [19]
*Trust*	High level of trust [46]	Trust concerns [19], perceived concern [43, 44]
	Positive first impressions [58]	Low level of trust [46]
	Improving data integrity [2, 40]	Unintentional misuse of the data [17, 40, 43]
	Data validity [58]	Concerns about misinterpretation of the data [17]
	Trust in data producers [40]	Open data can be reused for purposes they are not meant to be used for [19]
	Trust in the competence of the original investigator(s) (e.g. the original investigators’ membership in a Community of Practice; appropriate educational training of the original investigator) [58]	
	Transparent and honest attitudes of the original investigators [58]	
	Reputation of the researchers who collected the data	
	Trust in other researchers’ measurement [17]	
	Credible information availability [58]	
	Good intentions and ethics of the original study that produced the data (e.g. no commercial interests of the funder of the data; no apparent conflict of interest) [58]	
	Study's funding sources [58]	
	Existing evaluations of the data (e.g. many existing publications using the same data; large number of times the data has been reused and cited) [58]	
*Expected performance*	Expected performance [48]	Restrictions on use [50]
	Perceived (data) usefulness [17, 43, 44, 46]	Low perceived usefulness [46]
	Arrive to new findings [4], obtaining new insights [19, 48]	Potential waste of time [40]
	New scientific discoveries [2]	Effort may be wasted on flawed data [40]
	Being aware of the state of the art and not reinventing the wheel [48]	Negative reactions to data reuse [40]
	Feedback on the need for certain data and facilities [48]	Issue of how to access usable citation and attribution information [55]
	Reproducibility of key research findings (and also experimental methods) that could push science ahead [4]	Quality of reusing the data based on the context of the previous study [44]
	Allows collaboration across diverse groups [4]	Inappropriate management or mistakes in management [58]
	Limited resources encourages collaboration [48]	Original investigators' carelessness [58]
	Makes possible the testing of new or alternative hypotheses and methods of analysis [50], particularly when data are combined with other publicly available datasets [8]	Risk of misinterpretation based on inappropriate use of data [59]
	Supports studies on data collection methods and measurement [50]	
	Enables exploration of topics not envisioned by initial investigators [50]	
	Permits the creation of new datasets when data from multiple sources are combined [50]	
	Novel combinations of data [40]	
	Opportunities for co-authorship [40]	
	Shortening the research process (limited time and resources) [40]	
	Demonstration of data use value [40]	
	Recognition from peers [40]	
	Application of old data in new contexts [11]	
*Social influence and affiliation*	Social and affiliation factors [48]	Low social influence (e.g. from colleagues) [46]
	High social influence (e.g. from colleagues) [46]	
	Positive reactions to data reuse [40]	
	Social pressure [40]	
	Norms [40], including social norms (a researcher’s perceived belief of what other researchers think about data reuse practice) [17]	
	Perceptions of close colleagues [40]	
	Colleagues' recommendations to use the data [58]	
	Emotional connections/ interpersonal relations with the original investigators [58]	
*Effort*	Effort [48]	Perceived effort [43, 44] and expectancy that effort requirements will be high [46, 48]
	Avoidance of duplication [2]	Data not accessible [2]
	Expectancy that effort requirements will be low [46]	Difficult to locate and find the data [48, 59]
	The ease of data accessibility [10]	The difficulty finding or accessing reusable data [17]
	Findability of the data [54]	Difficulty to discover available and relevant data [40]
	Relevance and ease of use [58]	Data are not findable among hundreds of data repositories [2]
	Identifying the web API for dataset access [6]	Information overload: available data and information may become overwhelming [19]
	Efficiently create more opportunities without the burden of data collection and repetition of efforts [49]	Technology-related limitation (e.g., reluctance to use online databases because of complex user interfaces making data entry time consuming) [10]
	For accessing the registries—catalogs of datasets that allow researchers to indicate the existence of data without going through the process of adding their data to a repository and for accessing social surveys [57]	Investment of time and resources [17]
	Collaboration can be used as an alternative to overcome the problems of data reuse [47]	Too much time required to reuse the data [48]
		Low ease of use [48]
		Difficulty integrating data [17]
		Data are very difficult to interpret once separated from contextual information [57]
		Issues with understanding the context of the original research and, especially, how the data were processed [47]
		The lack of contextual information may make it difficult to analyze and interpret the data [19]
		Fragmentation of datasets: data are offered at many different places [19]
		Each discipline has its own terminologies which leads to heterogeneity [19]
		Existing open data portals barely provide visualization functionalities–users have to search for visualization tools themselves [19]
		Tools for using OGD are fragmented and hardly integrated [19]
		The lack of data about the data may hinder the adequate use of these datasets [19]
*The researchers’ experience and skills*	(Positive) past experiences with open data use [40, 48, 58]	Lack of experience with open data use [19]
	Familiarity with particular (comparable) types of data and areas of research and research trends [59]	Lack of familiarity of the use of the data [55]
	Data-gathering experiences [59]	The required skills to analyze datasets [48]
	Knowing that the data is available [59]	Complex skills that are required for the new approaches to data [54]
	Specific knowledge about who is working in what areas [59]	Costs associated to training potential data users [4]
	Knowledge of how to handle data [40]	
	Researchers’ ability to understand open data [17]	
	Formal training for researchers in finding, acquiring and validating data collected by others [17]	
	Knowledge gained through disciplinary training [59]	
	Education [43]	
*Legislation and regulation*	-	Legal restrictions [18, 50]
		Data sensitivity [2]
		Concerns about privacy [19]
		Concerns about national security and trade secrets [50]
		Unclear use conditions / unclear what ‘openness’ means (large variety of licenses) [2]
		Challenges related to data ownership and its effect on the easy and efficient retrieval of data or information about data [59]
*Data characteristics*	Interoperability [50, 54]	The nature of data (some datasets are easier to be reused than others) [40]
	Standardization of data [47]	Data quality [50, 55] (trust that data are what they purport to be) [50]
	Data exchange via a standardized communication protocol [54]	Data quality issues [19, 58], e.g. missing variables; errors and flaws in the data [58]
	Technical and software standards [50]	Poor data documentation [48, 58]
	Digital identifiers [54]	Changes to the data over time [19]
	Data documentation [17]	Inability to determine the quality of the data [40]
	Comprehensive documentation of datasets and how to access them [50]	Data heterogeneity [19] and inconsistency between datasets [48]
	Good documentation in the form of detailed information about methodology and measurements [58]	Inconsistent or lacking metadata [2]
	Provision of sufficient metadata [54, 56]	Lack of references to other qualified metadata systems [54]
	Accurate and relevant attributes of metadata [13, 54]	Inability to discern dataset content and suitability for analysis (e.g. due to lacking metadata) [40]
	Consistent metadata [2]	Lack of interoperability [2, 54]
	Data type [40]	Not using standardized protocols; not using well-known ontologies [54]
	Data quality: good quality, trustworthy data and data lacking errors [44]	Lack of data standards [48] and varying data formats [55]
	Data meeting standards of scientific practice related to objectivity and representativeness [59]	Varying standards about data gathering [55]
		Data is not machine-readable [54]
		Datasets requiring proprietary software to be opened [54]
		Lack of harmonization of data formats, processing, analysis and data transfer [18]
		Multiplicity of data types [13]
		Lack of awareness regarding existing standards for data citation [13]
		Lack of clear usage license [54]
		Data access fee [55]
		The large volume and size of the data [48]

## Open research data adoption: Thematic analysis

**Fig 2:**
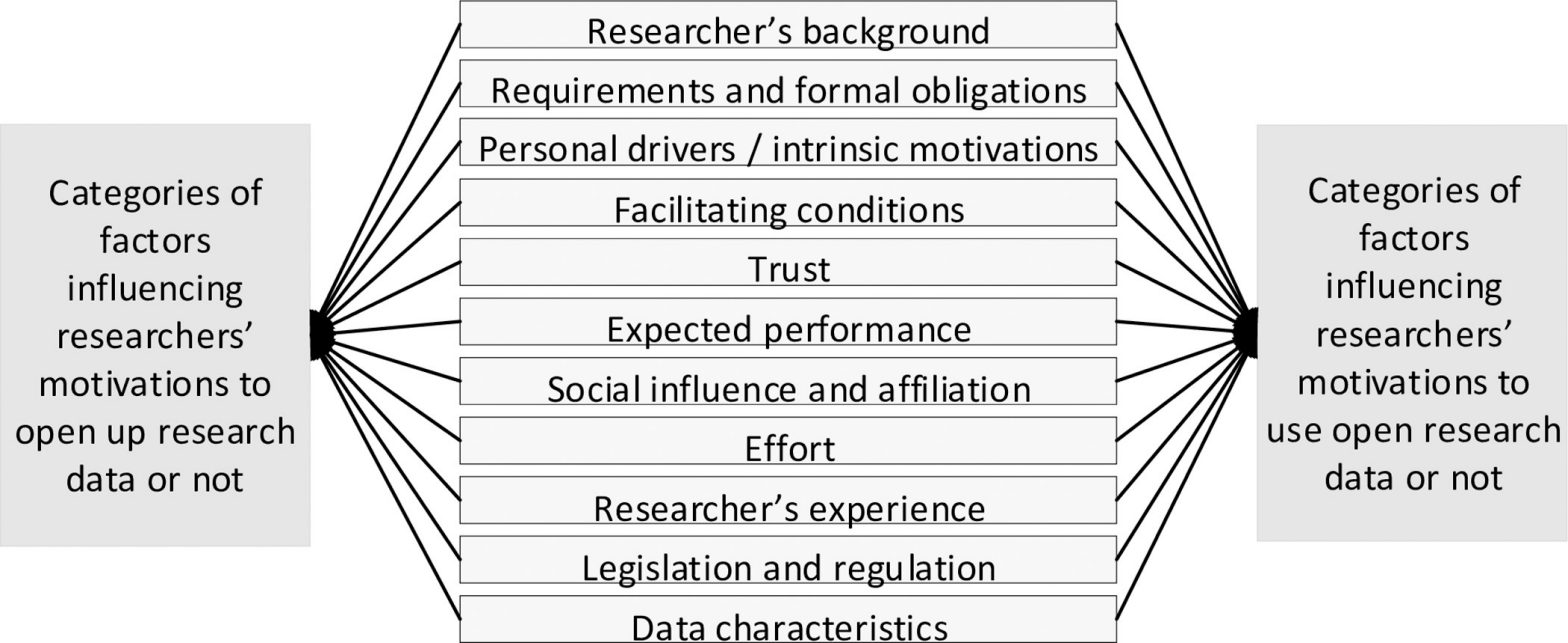
Categories of factors influencing whether researchers are driven or inhibited to share and use open research data.

This section focuses on the thematic analysis of the studies included in the literature review. The previous section provides insight into the factors driving and inhibiting open research data sharing and use. In this section, the categories that hold vital roles in open research data adoption are combined (Fig 2). Each of the eleven categories that the factors relate to are further detailed in the following section, followed by an overview of the categories and factors thereafter.

### Description of open research data adoption categories and factors

#### Researcher’s background

We found that various factors related to a researcher’s background altogether impact both open data sharing and use behavior. Such factors should be considered in relation to broader social, organizational, and cultural factors at play that influence people’s behavior. Research data sharing can be driven by disciplinary practice; organizational and academic culture and practice, and/or the researcher’s level of involvement in both research and teaching activities.

First, research data sharing is more common in certain disciplines than in that of others [11, 40]. It has been argued that disciplines such as genetic genealogy, atmospheric science, and oceanography have well-developed traditions of free and open access and robust databases, whereas disciplines such as wildlife ecology, medicine and many of the social sciences do not [56]. Others have argued that biology researchers tend to openly share research data more than medical/pharmaceutical-related researchers [47]. Likewise, political science researchers are more inclined to openly share compared to sociology-related researchers [45]. Various studies have found that certain research disciplines might have certain nuances, traditions, cultures, or “climates” that can altogether empower researchers to share open research data [17, 40, 43, 44, 56]. Whereas, a specific research discipline’s certain culture or habits might inhibit research data sharing. Yet, in the selected studies, there was no mention of disciplinary practices as an inhibiting factor.

Second, open research data sharing can be driven or inhibited by certain organizational culture [11], academic culture [47], a supportive data sharing culture [48], and organizational practices. In the literature, both cultural and organizational factors are namely mentioned as driving factors. This study argues that if both the culture and organizational practices are by default to not share research data openly, a researcher is less likely to openly share research data on one’s own. Both the organizational culture and practices might be related to disciplinary culture and practices, since disciplinary research has often been organized in different organizations (e.g. university faculties).

Third, the researchers’ involvement levels in both research and teaching activities altogether impact if they openly share their respective data. Researchers who only conduct research, in contrast to researchers who have time-consuming teaching obligations, are more likely to make their research data available to others [11]. Thus, the involvement in research and nothing else can be considered a factor driving open research data sharing. Whereas, the involvement in both research and teaching inhibits open research data sharing.

Fourth, some studies included in our review refer to demographic factors that differ for researchers who are openly sharing data to a smaller or larger degree. Such demographic factors by themselves do not explain researchers’ data sharing and use behavior. Yet, their occurrence differs for researchers who openly share research data compared to those who do not openly share research data. For example, Sayogo and Pardo [49] found that the probability of research data sharing among respondents from namely North American jurisdictions differ for both male and female researchers. In addition, research data sharing and use behavior is altogether more common in some countries than in others [17, 56] and non-tenured researchers are less likely to share their research data openly than tenured researchers [11]. Correlations between age and data sharing behavior are also found, although the findings are inconsistent. Tenopir, Allard [56] observed that older people (over 50) show more interest in sharing data and younger people are less likely to make their data available to others. Schmidt, Gemeinholzer [55] found that younger researchers (age 20 to 35) are more concerned about the impact of openly sharing research data compared to older researchers (age 51 and older). In contrast, da Costa and Leite [47] found that younger researchers are not less but more inclined to openly share their data, both due to their abilities in the use of technologies and to their interest in collaborating with researchers working on other research projects. It is likely that various intermediating factors impact the correlation between the factors of age and likeliness to openly share research data. In general, it should be stressed that demographic factors such as age, gender, country, along being tenured or not need to be viewed in the context of other broader social, organizational, and cultural factors that play a role in researchers’ decisions to openly share research data or not. For example: Enke, Thessen [10] observed that in general, researchers from Germany and Canada altogether often feel less willing to share research data than researchers from the United States or Europe. This difference might be related to socio-economic characteristics, current data sharing policies in place in these countries, [11] or cultural differences [40, 49]. Such factors have been examined only for particular countries in the set of studies included in our systematic literature review, coupled with the impacts and direction of such factors still need extensive research in the future.

With regard to open research data use, drivers found in the literature include: research discipline practices [17, 40], disciplinary climate (a sense of community and openness with other researchers affiliated in the same field) [17], the research climate [43], if data reuse is considered a prevalent practice in the researchers’ research community [44], existing traditions [40], and the sector the researcher works in [17]. Just like for openly sharing data, there might be differences in open data use behavior across researchers who have respective origins from different countries, along with older and younger researchers [17]—although such factors are not considered as drivers of openly sharing research data.

#### Requirements and formal obligations

Most of the factors found in relation to requirements and formal obligations concern the sharing of research data rather than the use of it. In data sharing’s context, both requirements and formal obligations relate to the increased pressure to release data [57]. These can be considered soft requirements, such as both the pressure and policies to openly share research data as defined by funding bodies, government agencies or journal publishers, existence of government directives, or encouragement by the federal government to create a robust data management plan. This category is different from the category of legislation and regulation (see Section Legislation and regulation) that is based on hard regulations such as government rules that forbid or mandate data releases, such as the European Union’s “General Data Protection Regulation” (GDPR) and the United States’ “Health Insurance Portability and Accountability Act” (HIPPA).

In the category of requirements and formal obligations, Fecher, Friesike [11], Kim and Adler [42] and Schmidt, Gemeinholzer [55] refer to the altogether impacts of funding policies and grant requirements, as funding agencies demand data sharing in return for (financial) support. As such factors drive researchers to openly share their respective research data. Occasionally, researchers receive research data from external agencies and use this data as secondary data for their respective research. Often, the external agencies provided them with the data under the condition that these agencies would also share the data openly with the public after a certain period (usually one year), and thus researchers considered this is a form of ‘automatically’ sharing research data openly [48].

A second factor related to requirements and formal obligations concern the requirements [41, 42, 47, 55] or even mandates [40] of scientific journals to openly share underlying research data when an article is published using that data. Also, openly sharing research data is driven by ethic codes [41] and the mandates for the creation of data management plans from federal agencies [56]. Generating data management plans forces researchers to think about what they will do with their data and requires an explanation if their data will not be published openly. Likewise, compliance with governmental directives can be a premise for opening up research data per Curty, Crowston [40].

Also, this study’s literature review specified university policies as a possible driver of openly sharing research data [48]. Equally, the policies of research institutes might play a vital role in the decision to openly share research data. For example, if a university or research institute mandates that all research data and code supporting the results described in a doctoral thesis are needed to be published openly or else one cannot complete the graduation requirement. Or when a university states that all research should be open unless the researcher explains why this cannot be done, this in fact might be a driver for sharing research data openly.

Factors inhibiting the open sharing of research data as identified from the literature include the possible loss of funding opportunities [13]. If the data is already openly available, there is thus no need to obtain funding to gather the data again. Furthermore, if the funders do not require researchers to openly share research data or if too many data policies apply, this has been said to inhibit research data sharing [55]. Namely as the latter might be confusing to researchers–thus having an adverse effect. Another inhibiting factor relates to the fact that study sponsors, particularly from industries, might not agree to release raw detailed information [8]. Companies might experience the risk of losing their competitive advantage if the collected data is openly shared [8].

In using open research data’s context, a factor that drives researchers involves the existence of policies that stimulate researchers to use available open research data [17] and whether researchers experience peer pressure [40]. Another driver not mentioned in the literature is researchers’ needs to use open research data for their job or a particular study. For example, when a particular question can only be answered using available open data. This driver is particularly present when it is difficult to obtain the data and when there are strong needs to answer a particular (research) question for which the available open research data is vital. The use of open research data is inhibited as many varying policies on both access and reuse across countries [2] that might in fact confuse researchers and thus result in reluctance to use open research data. What’s more, possible ethical bottlenecks might hinder open data use [18].

#### Personal drivers and intrinsic motivations

The third category of factors impacting both open research data sharing and use concern personal drivers and intrinsic motivations. Fecher, Friesike [11] refer to five-character traits influencing researchers to openly share their data: openness to experience, conscientiousness, extraversion, agreeableness, and neuroticism. The presence of higher or lower levels of such character traits within individual researchers can either drive or inhibit them to openly share their data. Also, scholars refer to both personal drivers [48] and a positive attitude toward data sharing [45] as vital individual drivers for openly sharing research data. Coupled with character traits, other drivers for openly sharing research data relate to either individual incentives [17] (e.g. wanting to learn about yourself [18], perceived behavioral autonomy [45] and self-efficacy to be able to share data [45]) or societal incentives (e.g. better informing society and fostering new learning processes [4]). Equal access to publicly-funded data can likewise be considered a driver by itself [4] as this offers individuals the opportunity to both better understand our social-physical world [50] and provides decision-makers with the vital facts needed to address complex and often transnational challenges [50].

Researchers might be driven to openly share their data due to strong beliefs. They might be convinced that all data generated with public money should be made public [10], especially when this data has reuse value for many years [52]. Researchers might both be personally committed to open data and to respond to requests from data users [55]. Also, they might have a strong sense of responsibility about both the dissemination and recognition of research results [55]. Research data should be accessible for multiple disciplines and for researchers from different disciplines [42]. This is expected to encourage both the validation and verification of research results [2, 56], along with enable falsification [11]. Open research data can help identify errors and discourage research fraud [8, 9]. The public can scrutinize the data in-depth by analyzing, processing and combining the data. Both opening up research data encourages multiple perspectives [8, 42], along with allows other researchers to explore newer data interpretations [17, 56], ask new questions [57], pursue newer lines of research [57], and test different hypotheses [42]. Thus, valuable resources can contribute far beyond their original analysis [9]. Opening up research data is not only beneficial for researchers, but also for society overall as sit provides a democratic scientific knowledge-sharing platform: “Open access increases the pool of information available to anyone not just scientists” [4, p. 466]. A lack of concerns about ethics and the commercial potential of data altogether contributes to more data sharing [48].

Opening up research data can be driven by the intrinsic motivation to facilitate comparisons between methods and sites [57], increase the knowledge in the field at-hand [17], move the field forward more quickly and easily [48], encourage economic development and spur innovation [2], identify synergies [11], accelerate scientific progress, [11, 17, 55, 57] contribute to the advancement of research [18, 42, 52], gain newer insights for data-driven research [19], and enable citizen science and encourage public activism [1]. The research data’s usability [48], the research community’s size [52], and the extent to which data is viewed as a vital asset [52] also altogether impacts research data sharing levels. Other factors include research’s improved discoverability [9, 17]; extending research from prior results [56]; a focus on best work via data availability [9]; the generation of new datasets, information, and knowledge when data from various sources is altogether combined [19]; educating researchers about the more consumer side of open data practices [17], and providing the opportunity to review works derived from the dataset [56]. Drivers that are not mentioned in the literature, but that may play a role include: enthusiasm, curiosity, joy, and moral obligation. Many drivers for openly sharing research data have been mentioned in the studied literature, while only few inhibitors have been mentioned: the fear that the data will only be reused by few [51], laziness [55], a negative attitude towards data sharing [45], and the commercialization of research findings [11]. If research findings are openly shared, the possibility of commercializing such findings becomes more limited.

Moreover, personal drivers for using open research data are identified from the literature. Researchers can be motivated to use open research data because of scientists’ beliefs and attitudes [40]. For instance, believing that it is fun to explore data [18], believing data reuse is good [44], individual willingness [40], open data use reinforces open scientific inquiry [50], encouragements of both analysis and opinion [50] or the promotion of new research [50]. Open data use is also driven by the belief that it might stimulate economic growth and the replication and validation of research [2] as it might enhance transparency and reproducibility of the scientific enterprise [40]. Using open research data might be impacted by researchers’ feeling of worth, namely the feeling that the time spent on data reuse is well spent [40]. Via open data use, research results may be replicated [11] that can advance researchers’ understanding in specific domains, such as health and disease [18], or in general. Other personal drivers for using open research data are that it: accelerates research [18], allows exploration of new interpretations of data [17], increases the knowledge in the field [17], because there is a strong intention to reuse data [17, 43] or because data being used enhances public trust and knowledge of the discipline at-hand [17].

In the studied literature, only a few inhibitors for using open research data are mentioned. Curty, Crowston [40] reflected that the altogether of researchers’ beliefs and attitudes on whether they will use open research data or not. Joo, Kim [17] also refer to attitudes, along with researchers’ perceived concerns. Finally, Yoon [58] refers to a negative first impression that might inhibit researchers from using openly shared research data.

#### Facilitating conditions

Facilitating conditions can drive researchers to both openly share their data and use open data shared by others. However, the inverse of this is that the lack of facilitating conditions can both inhibit open research data sharing and use behavior. Facilitating conditions mentioned in the analyzed studies about open data sharing concern the availability of infrastructure [17, 57], and more specifically, an appropriately designed (technological) infrastructure [50], appropriate information systems [47] and better ICT facilitation (e.g. the Internet hosts per person; percentage of computers per household; continued rate of growth of chip, storage, and network technology capacity) [50]. Wallis, Rolando [57] have detailed that researchers working in the hard sciences that have richer investments of funding, labor, scale, and infrastructure are in fact more motivated to openly share their data than those working in sciences where this is uncommon. Also, the lack of appropriate infrastructure inhibits openly sharing research data [57]. And open data infrastructures need to be sustainable, flexible and robust in the long-term as researchers are less likely to openly share their data if it is unclear whether the infrastructure enables long-term access to their data. Flexiblity allows for adaptation to the latest technological and other developments in society. The latter is a driver for openly sharing research data that we did not find in the studies selected for our literature review.

Another driver for openly sharing research data in the category of facilitating conditions concerns the availabilities of both large data repositories [13, 17, 41, 42, 47] and archives [13] in which researchers can store data. One could even consider this a critical factor, since without these storage facilities, the data cannot be opened up. Both grow storage and access capabilities should also have the ability to grow and still operate reliably and efficiently [2] as datasets in some domains can be extremely large. Other drivers for openly sharing research data include both continued and dedicated budgetary planning plus appropriate financial support [50], a short embargo period [52] and consent such as informed consent or contractual consent [11] for opening the data. While such support types are related to facilitating conditions, other support types are more related to effort (see Section ‘Effort’). With regard to funding, da Costa and Leite [47] argue that “adequate funding for the treatment and availability of data can generate savings in resources in future research funding” (p. 920). Moreover, when funding specifically for the management of research data is available, this might motivate researchers to openly share their respective research data [47].

Inhibitors for openly sharing research data are often found in the area of financial arrangements and budgets [50], and financial resources [11, 41]. For example, the loss of potential licensing revenue that would accrue to inventors of patentable discoveries has been considered as a financial barrier [1]. Also, inhibitors exist in terms of technical challenges [17, 50], such as limited openness of ICT tools which help in opening the data [1]. They may also be organizational, such as when institutional members resist change [1], when there are structural conflicts and managerial practices in the organization (e.g. security reasons, financial interest) [49] or when there is not enough time [48], for example, not enough time to organize the data [41]. Other inhibitors for openly sharing research data include the lack of a data repository [42], the lack of facilitating platforms [48], the lack of information systems to disclose research data in certain research disciplines (e.g. medicine) [47], difficulties with the communication of the open data results [1], the lack of tools to observe data metrics [54], a long embargo period [52], the perceived short reuse value [52] and science that can be considered ‘small’ (science that has less investment in funding, labor, scale, and infrastructure) [57].

Specifically in the contexts of both Kenyan and South African chemistry laboratories, Bezuidenhout [51] refers to inhibitors that inhibit research data sharing by researchers in low-resourced research settings. First, such researchers experience a lack of available resources, equipment and infrastructure that algother slows down the pace of research and that makes it even more important to only share research data openly once the related publication is out [51]. For instance, research data sharing is limited in this context because of a lack of power, older equipment, poor maintenance, a lack of technical support, a lack of ICTs, a lack of platforms, along with a lack of appropriate software for openly sharing research data [51].

About facilitating conditions related to the use of openly-available research data, various facilitating conditions-related drivers were identified. First, several drivers are related to technical aspects such as digital tools [4]. The potentials to involve more actors in data collection through citizen science platforms, unrestricted by physical or cognitive distance, has led to the facilitation of more data collection from various sources [4]. Other technical drivers for open data use concern the availability of an open data infrastructure [19], particularly a robust infrastructure for long-term usage [50], along with the availability of data repositories [17, 43, 44]. An initial large data repository can foster a culture of both data sharing and reuse [17]. Also, technical support might ease the process of open data use. For example, via the use of specialized software or programs [43]. A final technical driver includes the possibility to cite and attribute datasets, to foster a scholarly communication system that altogether allows for the identification, retrieval, and attribution of research data [13]. Drivers for using open research data in relation to facilitating conditions are organizational too. Both of these include the organizational environment [17] and institutional support [17, 43], such as any available assistance that researchers could acquire from their affiliated institutions or organizations, particularly technical or human help [17]. Also, human resources for questions are mentioned by Kim and Yoon [43], as they refer to advisors, data reuser groups, and data producers as human resources altogether for support.

Inhibitors for open research data use in relation to facilitating conditions mainly concern technical bottlenecks [18] and the functionality of the infrastructures and portals. Examples of the latter are the lack of the necessary infrastructure support for quick data analysis [47], the lack of approaches that offer both precision and recall when it comes to locating data for reuse [59], the lack of interaction support and tools, the limited availability of search options for open datasets, the lack of support for searching for data in multiple languages, the lack of support for data analysis functions, and the limited availability of functionalities related to interaction with other open data users or data providers [19]. The lack of availability of the data itself [19], heavy reliance on the methods and techniques data producers employed to obtain, organize, and code the data [40], along with doubts about the long-term availability of the infrastructure [19] are other inhibitors for using open research data.

#### Trust

Trust can be a very impactful driver and inhibitor for open research data sharing [17, 52]. In the literature review, several aspects of trust that drive openly sharing research data were identified, namely the trust of peers and society in general in the research findings, open data users’ trust of individual researchers, researchers’ trust in their own research findings, and individual researchers’ trust in the open data portal and long-term preservation of their data. First, researchers may openly share their data to make them transparent and to show others that includes other researchers and society at large, that they can trust the research findings, as this might lead to greater credibility of the research findings [42]. Altogether, transparency of study results [10], research methods and processes [18] can enhance the trustworthiness of the research results and drive open data sharing. It can also increase the reproducibility of the research results [48]. It has also been found that data availability provides safeguards against misconduct related to data fabrication and falsification [56], since this makes it easier to interpret the data [59]. Second, if researchers better understand what users may or may not do with data in online data repositories, their drive to open up their data may be enhanced [2]. Researchers often want to have a say in data use [11] and want to have the ability to place conditions on data access [56], such as data security conditions [11]. Such conditions lower the likelihood of misconduct with the data and enhance a researcher’s trust in the user of the data. Furthermore, the lower the privacy risks, the lower the risk for trust issues [18]. Third, researchers might trust their own study’s conclusions more when multiple users reach the same conclusions using the same data. Thus, ensuring the validity of the data by multiple users can be considered another driver for openly sharing research data [1]. Fourth, another factor that might drive researchers to openly share their data concerns the trust of individual researchers in the open data portal and particularly, in the data’s long-term preservation. Researchers publish their respective data on a certain open data portal with the idea that the data will be available in the long-term, and with the assumption that potential users will be able to easily access their respective data. Per Tenopir, Allard [56], well-managed, long-term preservation helps retain data integrity. Openly sharing research data can then be considered good management of data integrity in time [2].

Trust-related inhibitors for openly sharing research data include the fear of losing control over the data, the fear of: possible unethical data use (includes both data misinterpretation and misuse), data’s commercialization, the fear of harm to the researcher, the level of trust in the data of other researchers and the knowledge about the data user, and losing a valuable resource that could have been used to obtain other data. First, the loss of control [10], such as the lack of control over the scientific findings and conclusions derived from the original data that a researcher shared, inhibits open data sharing [1]. As once research data has been published online, the data can be copied, changed, and published elsewhere in various forms. Second, there might be issues regarding ethical responsible use of shared data [49], and possible data integrity concerns [17]. Someone might draw the wrong conclusions [10], for instance, as the result of data’s flawed interpretations [11, 52, 55] or even misinterpretation and misuse of the data [19, 41, 49, 52, 55]. And possible data misuse incidents may take place [52]. Researchers might also fear the commercial or competitive misuse of the data [11]–causing potential harm to the data publisher’s reputation [11, 19]. Third, the difficulty in establishing trust in others' data inhibits openly sharing research data [57]. If a researcher has little trust in others’ data, the researcher might assume that others might too have little trust in his or her data if it was openly shared that altogether demotivates the researcher to do so. Fourth, the level of knowledge about the data user [11] has been found to influence the trust a researcher has in the ethical use of his or her data when it is shared openly online. If the intent of the data user is unclear, this can thus inhibit data sharing [11]. The more knowledge the researcher has about the user of his or her data, the more he or she may trust this person and the use of the data. Fifth, by openly publishing their respective data, researchers might fear losing a valuable resource that could have been used to obtain other data. Wallis, Rolando [57] refer to the “gift culture of scholarship”, meaning that researchers sometimes exchange valuable data through a trusted relationship with other individual researchers. This means that if they have no data to share with other individuals, they might not obtain valuable data from them. Sixth, the lack of trust in the data portal may inhibit open research data sharing, for instance as supplementary information and laboratory sites are transient [8]. Finally, one factor was missing from the overview: the lack of trust of researchers in their own respective research findings. This factor was not mentioned, but it is strongly assumed that it might be a vital inhibitor for openly sharing research data.

Trust is not only vital in sharing research data’s context, but also in the context of using it. Higher levels of trusts are linked with increased use of open research data [46]. In the literature, seven aspects of trust that drive researchers to use open research data were identified. One driver is the will of a data user to improve data integrity [2, 40]. Open research data might be used to investigate if research is both reproducible and trustworthy. A second trust-related driver for using open research data concerns the trust that a data user has in the data’s producer [40]. Researchers might be more motivated to use a certain open dataset if they trust the dataset’s producer or provider [40]. Trust in the dataset’s producer may increase when this person is altogether honest and transparent, received appropriate educational training, and is member of a trusted community [58]. The reputation of the researcher who originally collected the data is thus vital [58]. Although this was not mentioned in the literature, expected was that trust in the data producer too increases that the potential data user knows the researcher who collected the data or the organization that provided the data. This factor is related to the “social influence and affiliation” category. Moreover, as a third influencing factor, open research data use is impacted by the sources that funded the study [58]. If the study’s funder has both no commercial interests and lacks apparent conflict of interest, this thus increases the researchers’ willingness to use open research data [58]. A fifth trust-related driver for using open research data concerns the availability of credible information about the study [58]. For instance, when both the metadata and related documentation explains the data collection procedures. This factor is related to the “data characteristics” category. Sixth, open data use might be driven by a data user’s trust in the researchers’ measurements [17] and thus in the data itself. Data quality, data validity, attribution and soundness of contextual information have altogether become critical factors influencing researchers’ motivations to use open research data. A positive first impression of the dataset is vital in making a decision about if the researcher will use an openly available dataset or not [58]. This factor is strongly related to the aforesaid “data characteristics” category. Finally, the data’s existing evaluations increase the likelihood that a researcher will use open research data [58]. For example, when many articles have been published using the same dataset or when a dataset has been reused and cited often, this thus increases trust in the data [58].

The use of open research data is inhibited by trust-related concerns [19, 43, 44, 46], such as concerns about the aforesaid possible data misinterpretation [17] and unintentional misuse [17, 40, 43]. As data users might unintentionally make mistakes in both data interpretation and use. And that open data can be reused for unintended or unexpected purposes [19]. Inhibitors for using open research data that were not explicitly mentioned in the studied literature are the lack of trust in the producer and provider of the data, the lack of trust in the methods used to collect the data, and the lack of trust in the data itself. Such new factors are added to the factor overview.

#### Expected performance

There are many drivers for openly sharing research data that relate to the expected performance of researchers. As by opening up their data, they expect to perform better [11, 48]. The performance-related drivers found are as follows: First, researchers are driven to openly share their data both for possible collaboration and network opportunities. For example, openly sharing data creates ample opportunities to participate in new international projects, widening local scientists’ networks [4], and allows networking with other scientists for various interdisciplinary studies [10]. And data sharing enhances the potential for collaboration among scholars with similar research interests [41, 48]. Second, opportunities to obtain research data via professional exchanges can further drive researchers to openly share their data [11]. Third, openly sharing data might increase scientific efficiency [4], since it is an effective way to both archive and preserve data [2]. Fourth, openly sharing research data can enhance the capacity to solve specific problems. For example, via interactions with other actors, research agendas could be better guided towards solving problems that affect a specific group [4], along with cheaper solutions to societal problems might be found [4]. Furthermore, by opening up their data, researchers can help make local problems both become more visible and better communicated [4], coupled with other people can offer input to develop final solutions [4]. Fifth, researchers might be driven to openly share their data when appropriate reward structures are put in place [13, 50] and especially when they are recognized for doing so [11, 47]. This recognition can be both institutional and professional in nature [41]. Sixth, openly sharing research data can increase both researcher’s visiblity and his/her research. Formal citation and receiving proper data citation credit [13] can be considered one form of recognition. Another form is the acknowledgement of the dataset's originator in terms of appreciation (e.g. co-authorship on publications, formal acknowledgement of the data providers, or opportunity to collaborate with others) [49]. Recognition can too be established in the form of citations and visibility of research, researchers and research institutions, such as systematic visibility of the data source [50], increased visibility and relevance of research output [17, 47], an increase in the researchers’ visibility in the community [10, 48], increased visibility of the institution in which the research was carried out [47] and altogether increased citation rates of datasets and publications [8, 9, 40, 41, 48]. Thus, openly sharing research data is a robust approach to demonstrate the value of a researcher’s own accomplishments [57]. Seventh, data may also be shared openly because of perceived career benefits as a result [42]. This factor is strongly correlated with the aforesaid reward structures and other recognition forms. Openly sharing research data can be considered one aspect of professionalism, namely to build upon codes of conduct and ethics of the scientific community [50]. A specific example of a career benefit driving researchers to openly share their data is the opportunity to publish the research results in journals of great international prestige [47]. This factor is too related to the category of ‘requirements and formal obligations’. Eighth, openly sharing research data can lead to improvements in terms of data scrutinization, comprehensive analyses, hypotheses testing and data quality. When comparable datasets are highly available, this thus enables comprehensive analyses [10]. These comparisons may improve the understandability and quality of the data, since multiple researchers may then work with and scrutinize the data. Both the review and quality improvements are drivers for openly sharing research data [11], along with additional evaluation capability. For example, other researchers might test the data and hypotheses [2], allowing them to confirm the findings of the original publication or to test different hypotheses [41]. Ninth, data might be shared openly because researchers may promulgate technology as a basis for others’ research [57]. Tenth, researchers openly sharing their data could result in greater returns of public investments in research [50]. For instance, wealth might be generated via a proactive downstream commercialization of outputs [50]. Finally, research data may be shared to improve decision-making on a particular topic. Researchers can provide evidence to support an analytic framework and related decisions [42].

In relation to performance, researchers might feel inhibited to openly share their data for the following reasons. First, they may not want to openly share their data as they might fear the loss of control over unpublished data in publicly-accessible online databases [10] or their research products [52]. They might be concerned about losing an advantage in their research area [17]. Second, researchers might fear receiving no credit or reward for data sharing [13, 50, 52, 55, 59]. Someone else might publish using their data with no returned reward since there is no system of acknowledgement [13]. As stated by Mooney and Newton [13], references to the name of the data creators and publishers are scarce or not prominently featured (mostly, there are only references to the dataset title). Data is often not cited properly [52], and as an enhancing effect, citations of research data are boht insufficiently recognized and valued. Thus, there is a lack of compensation for the required effort from researchers [47]. Both current incentives and merit systems, which lack sufficient rewards for researchers, inhibit open research data sharing [49]. Third, researchers might not openly share their data because they fear that they will be possibly deluged with requests for assistance [8]. Fourth, researchers might be inhibited to openly share their data because they fear they will decrease their own competitive advantage [8]. Openly sharing research data can also result in a perceived career risk [42], related to losing funding opportunities [13], losing potentially profit-making intellectual property [8], losing commercialization opportunities [42], and missing out on future publishing opportunities [8, 13, 41]. The latter especially concerns the fear of results scooping additional analyses that researchers have planned for the future [9, 48]. Other concerns involve protecting the researchers’ right to publish their results first [57]. Such inhibiting factor is strengthened by the fact that most academic incentive systems favor publishing articles over publishing data [47, 57]. Researchers prefer to publish their results before openly sharing their data [55]. Furthermore, researchers might fear losing information trade-in-kind offers with other labs [8]. Researchers might lose the abilities to privatley barter data privately that thus creates a disincentive for openly sharing research data [57]. Additionally, researchers might be afraid of criticism of their data or analyses [10]. Investigators might worry that other researchers will find errors in their respective results [9, 48] that might harm their reputation [52]. By openly sharing research data, the original conclusions might be challenged by a re-analysis, whether due to the original study’s possible errors, misunderstanding or misinterpretation of the data, or simply more refined analysis methods [8]. This relates to the fear that researchers need to both spend time reviewing and possibly rebutting future re-analyses [8]. Finally, openly sharing research data might be inhibited when researchers believe that data has limited usability value to others [48]. In the context of research into data sharing in developing countries, it has too been stated that researchers might not openly share their data because they are concerned that if data would be released it would not be reused by their fellow international peers [51]. The premise is the fear that the equipment used to produce the data is not as advanced than that of researchers in developed countries [51].

Also identified are various performance-related factors that impact open research data’s use. Drivers for open data use include: perceived usefulness, the ability to gain new insights and push science forward, collaboration across divers groups, enabling the exploration of topics not envisioned by initial investigators, testing new or alternative hypotheses and methods of analysis, coupled with making new data combinations and shortening the research process. First, the researchers’ opinions about whether a particular dataset can be useful for their purposes may drive them to use it [17, 43, 44, 46]. Perceived usefulness might be influenced by the second driver, namely the ability to arrive at new findings [4] and obtain new insights [19]. With open research data, researchers become more aware of the state of the art and the need for certain data and facilities, rather than somewhat ‘reinventing the wheel’ [48]. Reproducing key research findings and experimental methods could push science forward [4] that thus enables the application of old data in new contexts [11]. Third, when a researcher finds out that another researcher has openly shared data on a topic that is of interest to both of them, they might start collaborating on the use of the shared data. Thus, open data use allows proactive collaboration across diverse groups [4], especially when resources are limited [48], and offers more opportunities for co-authorship [40]. Thus, peers can give each other recognition for their efforts [40]. Fourth, using open research data enables the exploration of topics not envisioned by initial investigators [50]. Fifth, using open research data makes it possible to test new or alternative hypotheses and methods of analysis [50], namely when data are combined with other publicly-available datasets [8]. Thus, open data use permits the creation of new datasets when data from multiple sources are combined [50], which can lead to novel combinations of data [40] and new scientific discoveries [2]. These demonstrate the use value of data [40]. Finally, researchers are driven to use open research data in order to shorten the research process [40]. This is especially vital when researchers are limited on both time and resources.

Inhibitors for using open research data include the existing restrictions on data use [50], so that they cannot be used to perform as desired. The data might too be perceived as not useful [46] with the risk that the effort might be wasted on flawed data [40] and thus a potential waste of time [40]. As another performance-related factor, researchers might be inhibited to used open research data because of negative reactions to data reuse [40]. And it can be difficult to access information needed to cite the dataset and attribute the data producers [55]. Finally, the quality of reusing data is per the study’s context in which the data was created [44]. If data had been managed inappropriately or mistakes have been made this thus reduces the researchers’ motivation to use open research data [58]. Likewise, carelessness on the part of the original investigators to manage the data well [58] and possible misinterpretation risks per inapproriate data use [59] might altogether inhibit open research data use.

#### Social influence and affiliation

The analyzed studies too refer to social influence and affiliation as drivers plus inhibitors for both sharing and using open research data. Drivers for sharing open research data namely reflect social responsiveness, perceived normative pressure, standard social norms, subjective norms, pressure by journals, peer pressure, attitudes about data sharing, world-wide attention to the need to share and preserve data and codes of conduct, and related normative standards of professional scientists and their respective communities. Arza and Fressoli [4] have stated that social responsiveness is a factor that can drive researchers to share their research data openly. Both Kim and Adler [42] and Harper and Kim [41] have referred to the perceived normative pressure and standard social plus subjective norms, respectively. Normative pressure can relate to pressure by journals [41], as mentioned in the section “Requirements and Formal Obligations” section. Zenk-Möltgen, Akdeniz [45] refer to the perceived social pressure to share data with others. Social influence, such as peer pressure [8] can be a driver for sharing research data. For example, when the norm is not to share data openly or when a supervisor or colleagues simply tells you not to share your research data openly. For other influencing factors concern attitudes about data sharing [17, 42], there has been more worldwide attention to the needs to both share and preserve data [56]. Finally, there are the codes of conduct and related normative standards of professional scientists and their respective communities [50].

For the “social influence” category, the only inhibitor for openly sharing research data mentioned in the literature is the an open sharing-like culture [49]. Sayogo and Pardo [49] have stated that with regard to culture, academic promotion is tied to publications and not weighed much on sharing research data that thus altogether results in researchers prioritizing the publications of articles instead of data. Other possible social inhibitors for sharing open research data may relate to the identified drivers. For example, researchers might perceive normative pressure from their organization or colleagues not to openly share their data, as they may need to prioritize other tasks, such as teaching. Other inhibitors not identified in the literature but considered to be vital include standard social norms and subjective norms not to openly share data, along with possible negative attitudes toward data sharing.

In the “social influence” category, the literature refers to similar constructs that impact if researchers use open research data compared to their open data sharing behavior. For instance, Curty, Crowston [40] state that the factors driving researchers to use open research data include social pressure, perceptions of close colleagues, along with positive reactions to both data reuse and norms. For instance, colleagues might recommend researchers to use the data that can increase their respective motivations to do so [58]. And having an emotional or interpersonal relation with the original investigator was identified as a driver for researchers to use open research data [58]. Finally, Joo, Kim [17] refer to the driver of “social norms” (i.e. a researcher’s perception that other researchers think positively about data reuse practices).

The aforesaid examined literature mentions one social influence-related inhibitor for using open research data, namely the low social influence, for example, from fellow colleagues [46]. We hypothesize that other social influence-related factors might also inhibit open research data use such as both the social pressure and perceptions of research supervisors not to use open research data. Coupled with the perception or perceived norm that other researchers are not using open research data, negative reactions to data reuse and a researcher’s perceived belief that other researchers think negatively about data reuse practices. With all these in mind, such inhibitors need to be examined further in future research.

#### Effort

In open research data’s context, perceived effort is believed to influence researchers’ intentions to openly share their data and to use data that others have openly shared. This study’s analysis of effort-related factors have shown that researchers are driven to openly share their data since this prevents the duplication of work [2, 41, 48, 57]. The work can be used as a source for researchers to consult when considering how to build upon existing studies [42], so that data sharing can thus accelerate scientific progress. As not having to recollect data also means that openly sharing data reduces research costs [17, 41, 42] and thus saves time involved in the data collection process [41, 48]. Ultimately, this means that there is more efficient and optimized use of resources altogether [1, 8, 9, 48, 56]. As researchers are namely driven to openly share their data when they expect that it will be reused [40] and thus lead to increased data use [9]. What’s more, organizational support for data management is found to both reduce effort and drive data sharing [49]. Research data sharing can be stimulated when tailored data management approaches and institutional models are used that meet the researchers’ needs [50]. Previous research has found that when data is already cleaned, processed, refined and analyzed during the research instead of after the research, this thus increases the researchers’ willingness to openly share their data [52]. The fact that anyone can access the data and contribute to it may improve the quality of the research [48]. Also, it has also been stated that quantitative analytic work can motivate researchers to openly share their data, in contrast to qualitative work [42], as it is found that preparing qualitative research data for sharing requires more effort. Altogether, the use of software, equipment and data repositories can reduce the effort needed from researchers in openly sharing their data [47]. Other effort-related drivers for openly sharing research data include having assistance with data management across the data lifecycle [56], technical support [11], being able to identify the web Application Programming Interface (API) for dataset access [6] and adapting the query-result parser to distinguish between invalid UIDs, datasets that have been released, openly sharing parts of a dataset rather than to share the whole dataset [59] and datasets that remain private [6]. Finally, previous research has found that if researchers were not involved in the data collection themselves (e.g. when another researcher or external institution took care of this), researchers were more motivated to openly share the data [48].

The effort or perceived effort of openly sharing research data has been considered an important inhibitor [11, 41, 42, 47, 49]. Sometimes this required effort concerns manual effort [6] and this may require a large amount of work [52]. Several effort-related inhibitors for openly sharing research data relate to the required individual investment needed to both preserve and manage data [57] that includes time investment (i.e. amount of time researchers would have to invest to get the data ready to share) [8, 10, 11, 49]. To enable open data sharing, researchers might need to structure the dataset following a particular standard [47, 55], to describe the data more thoroughly than required for the original research [47] or to properly document the data so that it becomes reusable for other researchers [55]. Allowing for discoverable, reusable data from the long tail is emerging as a major challenge [57]. The efforts needed for the formatting, documentation, and release of the data inhibits research data sharing [8, 9], and these efforts appear to be higher for qualitative analytic work compared to quantiatative analytic work [42]. Effort can be technology-related too. For instance, researchers may be reluctant to use online databases because of complex user interfaces that make data entry time consuming [10]. Opening up research data can be complicated and thus hinder data release [8]. Other effort-related inhibitors for openly sharing research data include issues with the quality of the open data platforms and their credibility [1]. Especially with the lack of acknowledgement for the researchers’ effort [10], the experience that conveying information to the public is not always straightforward [1], along with the possible issues with authorship and with gathering permission from all partners involved in larger collaborations [48].

With regard to open research data use, this is driven by the factor that it may prevent the duplication of research data [2], as researchers can efficiently make use of more opportunities for data use without the burden of data collection or repetition of effort [49]. Likewise, researchers are more motivated to use open research data when they expect that effort requirements will be lower [46] and the ease of accessing open research data drives researchers to use such data [10, 57]. Also, motivations are increased when it is easy to find data [54] when the relevance of the data is clear [58], along with when the data is easy to use [58]. What’s more, researchers are more driven to use open research data when they can identify the web API for dataset access [6]. Finally, when researchers experience issues with open data use, collaboration can be used to overcome such issues [47].

Effort or perceived effort can inhibit open research data use [43, 44, 46, 48]. As sometimes the data is not accessible [2] that thus both naturally and immediately blocks the possibility to use it. And sometimes the data might exist, but cannot be found among hundreds of data repositories [2]. Thus, it can be difficult discover any available and relevant data [40] and the available data and information may become overwhelming [19]. Datasets might also be fragmented since they are offered at many different places [19]. Such difficulty might be in locating and finding reusable data [17, 48, 59]. The search for data requires researchers to invest time [17, 48] and resources in their data search [17], without knowing in advance if the time spent is wasted or useful. Researchers might be inhibited to use open research data because of low ease of use [48] that was possibly caused by technology-related limitations, such as their reluctances to use online databases due to complex user interfaces [10]. Once data has been found, it might be very difficult to both analyze and interpret since it is often separated from contextual information [19, 57], namely contextual information about how the data were processed [47] or due to appropriate metadata is lacking [19]. Tools to use such data are often both fragmented and hardly integrated [19]. Such factors too complicate the integration of multiple datasets [17]. Finally, open research data use is inhibited due to complex terminology heterogeneity (each discipline has its own terminologies that leads to heterogeneity) [19] and due to a of a lack of tools provided with the data (e.g. visualization tools that data users need to look for themselves) [19].

#### Researchers’ experience and skills

The identified literature shows that both experience and skill-related drivers for openly sharing research data include having access to data specialists [47], the possibility of data management consultation [52], the mastering of data management skills by researchers themselves [49], researchers having knowledge of metadata and its practices [41], along with researchers’ belief that open research data may be useful for training or educating students [42] and new researchers [8, 9, 50, 56]. It was also found that a researcher’s experience with openly sharing research data and his or her satisfaction with previous data-sharing experience(s) might be a driver for data sharing behavior [45, 48]. As other possible successful stories of other researchers openly sharing research data might too drive researchers to openly share their data, this factor was not identified in the studies selected for the literature review.

In contrast, a lack of skills, knowledge and expertise altogether inhibits openly sharing research data [11, 51]. Underlying this might be the inhibitors of a lack of data management skills and a lack of knowledge about metadata and its practices, although this was not explicitly mentioned in previous research. Other inhibitors that were not identified in the literature but that we believe might inhibit openly sharing research data concern a researcher’s lack of experience with openly sharing data, a researcher’s dissatisfaction with previous data-sharing experience(s), along with the dissatisfaction of other researchers (e.g. colleagues) with openly sharing research data. Negative experiences might result in reluctance to openly share research data.

Open research data use is driven by two main experience and skill-related factors. First, researchers who have positive past experiences with open data use might be more motivated to use open research data [40, 48, 58]. As they might already be familiar with what data is available [59] and find this data useful, have experience with collecting such data [59] and have knowledge of how to handle data [40] that altogether could save them time in finding and using data relevant for their own research. Especially having knowledge of particular (comparable) types of data and other research areas/trends, along with having specific knowledge about who is working in what areas can drive open data use [59]. Second, a researcher’s education [43], a researcher’s ability to understand open data [17] and formal training for researchers in finding, acquiring and validating data collected by others [17] can drive the use of open research data. Zimmerman [59] refers specifically to the usefulness of knowledge gained via disciplinary training [59].

Experience and skill-related inhibitors for using open research data can altogether be divided into three main factors. First, open research data use might be inhibited due to the lack of experience with open data use [19] and the lack of familiarity of such data use [55]. Second, researchers might be less motivated to use open research data when they lack the required skills to analyze datasets that can be quite complex in nature [48, 54]. A third inhibitor identified in this category both concerns and the costs linked with training potential data users [4]. Other factors that were not identified in the literature, but that might inhibit the use of open research data include a lack of education, an inability to understand open data, coupled with a researcher’s dissatisfaction with previous open data use. Such inhibitors are closely related to the experience and skill-related drivers for open data use, along with often concern either the existence of a certain skill or positive experience (drivers) or the lack thereof (inhibitors).

#### Legislation and regulation

In the context of open data, both legislation and regulation can either drive or inhibit researchers’ open data sharing and use behavior altogether [48]. As both legislation and regulation-related drivers for openly sharing research data include an established clear and transparent data policy [53], data sharing policy [11], journal policy [11, 42] and/or formal organizational policy [56]. It is especially useful when policies concerning data management exist across the whole data lifecycle [56]. Other drivers include support from national and local governments in terms of policies, programs and management practices [50], national laws and international agreements that stimulate data sharing [50], regulatory pressure [17], and legal and policy requirements that concern, for example, the significance of citation, legal agreements, statements of use, conditions of use, and approval for reuse [49].

With regard to legislation and regulation, openly sharing research data may be inhibited by legal rights and restrictions [2, 19, 49], along with other legal issues [10]. Data sources might be copyrighted such that the data subsets cannot be freely shared [8, 11]. Another issue related to licensing terms [50] is that one must choose from a large variety of licenses that could be confusing [2] to individual researchers. Researchers might consider licenses a burden [55], they might have concerns about having too restrictive licenses [55] or might experience difficulties in understanding licenses [55]. The law prohibits publication of certain data types [19]. And researchers might not be allowed by law to openly share their data due to certain intellectual property right issues [13, 17, 55], restrictions on use for private intellectual property rights [50], along with the fear of potentially violating property rights and other concerns such as those involving the legal liability for data or release of data [55], such as intellectual property or patent issues [10]. For some data, there might also be priority rights for publication [11]. Furthermore, ownership [11, 50, 59], the right of use [11], confidentiality [10, 11, 42, 55], and contracts with industry sponsors [42] are impactful inhibitors for data sharing. As data might also be sensitive [17, 19] or contain personal information that leads to privacy-related concerns [11, 17–19, 41, 42], namely as the sharing of privacy-sensitive data is prohibited by law. Data can be anonymized, but anonymization techniques cannot guarantee that individuals will not still be identified using certain re-identification techniques [60]. What’s more, privacy and the protection of trade secrets [50] can too be solid premises for not openly sharing research data. Another inhibitor concerns the different levels of security: public access may negatively impact national security [1, 50]. Coupled with datasets are sometimes created by multiple organizations with different levels of security, different policies, and different laws with which they must adhere to. Thus, all parties then need to give permission for the disclosure of the data [19]. Finally, informed consent agreements might not obviously cover subsequent uses of data and de-identification can be thus complex [8] that likewise inhibits openly sharing research data.

In the “legislation and regulation” category, not identified were any drivers for using open research data. There is no such thing as the use of open research data forced by regulation or legislation. At the same time, there are various legislation and regulation-related inhibitors for open research data use also referred to as “legal bottlenecks” [18]. These include the sensitivity of the data [2], concerns about violating privacy when using such data [19, 50], legal restrictions related to national security and trade secrets that could further complicate data use [50], challenges related to data ownership [59], and unclear conditions for data use, such as confusion about what is and is not allowed under a specific license [2].

#### Data characteristics

The last category, data characteristics, concerns the research data’s very nature. With the variety of methodologies, theories and research approaches altogether used and applied in different disciplines, unequivocal is that data is diverse in its domain, volume and type and may consequently be more or less difficult to use. Thus, the analyzed studies suggest that data characteristics might in fact be linked with researchers’ willingness to both share and reuse data.

With regard to data-related drivers, there are many factors that make it more likely that researchers will openly share their data that include: having effective data quality controls in place [50], good management practices [11], the use of dataset identifiers such as DOI [6], appropriate data documentation and metadata [11], along with following metadata standards [11] and formatting standards [11]. Furthermore, the chance of research data being shared increases when the data is in an easily digestible and appropriate form [52, 53] and format [52], when it is interoperable and complies with international agreements on interoperability [11, 50], along with when it does not involve human subjects, such as medical research patients [47]. Also, when data is sufficiently secure and when there are tools and applications for its use, openly sharing the data is thus more likely [11]. Cragin, Palmer [52] have found that researchers are more likely to share data that result from quantitative research than that from qualitative research. This might be caused by the increased likelihood of qualitative research to contain both privacy-sensitive information and the increased effort required to remove sensitive information from qualitative data compared to that of quantitative data. Finally, scholars in general have stated that the more data is produced [50] and stored [11], the more data is shared.

Various data-related inhibitors for openly sharing research data are interdependent with the drivers, since these are often the other side of the same coin. For example, while the use of data standards drives research data’s open sharing [e.g., 11], the lack of data standards inhibits research data sharing [10, 55]. Issues with data standards and protection inhibit research data sharing [49]. And while quantitative data collection increases the likelihood that researchers openly share their data, qualitative data might be considered an inhibitor for openly sharing research data [47]. Other inhibitors include inconsistent metadata [57], biased data [19], and other problems related to the mobility of data (i.e. data that is challenging to be thus moved to other facilities) [57]. Also, there might be possible quality issues [10, 11, 19, 49] and ones related to both local context and specificity, such as the specificity of purpose, events, and/or methodology and the duration of research [49]. What’s more, data might be too sensitive to share openly [47], such as when privacy issues are encountered [47], or the data format and form may not be appropriate for data use [52]. The data’s size may be too large to share the dataset [52] or may make it more difficult to share such data [48, 55].

Many of the aforesaid drivers and inhibitors too play a role in the decision of if to use open research data. In the analyzed studies, found was that the use of open research data is driven by appropriate data documentation [17] and namely comprehensive documentation of datasets and the approach to access them [50], along with the and documentation of both the methodology and measurements used to collect the data [58]. Metadata—data about the data—also plays a vital role in driving researchers to use open research data. The likelihood of researchers using open research data increases when datasets are accompanied by sufficient metadata [54, 56]: by accurate and relevant attributes of metadata [13, 54] and by consistent metadata [2]. Another driver for open data use concerns the data’s interoperability [50, 54], its standardization [47], the exchange of data via a standardized communication protocol [54] and the available technical and software standards that can be used to analyze the data [50]. An example of standardization in the open data use’s context concerns the use of digital identifiers [54] that ensures that datasets receive a unique identifier so that they can more easily be both found and cited. And more researchers are more driven to use open research data when the data is of good quality, trustworthy and lacks errors [44] and in general when it meets the standards of scientific research concerning objectivity and representativeness [59].

Data-related inhibitors for open data use concern issues with data quality [19, 50, 55, 58], such as missing variables, along with errors and flaws in the data [58]. This relates to the data users’ trust that the open data are what they purport to be [50] that is also related to changes to the data over time [19]. When a researcher is unable to determine data quality, this hinders or even blocks the use of the data [40]. Difficulties with determining the quality might be caused by poor data documentation [48, 58], data heterogeneity [19], inconsistency between datasets [48], inconsistent or lacking metadata [2], coupled with the inability to discern dataset content and hence suitability for analysis (e.g. because of a lack of metadata) [40]. Researchers might also experience a lack of references to that of other qualified metadata systems [54]. Likewise, open research data use might be inhibited by a lack of interoperability [2, 54]. For instance, the likelihood of using open research data decreases when the provided data is not machine-readable [54], when the data is provided not using standards [48, 55] and not using standardized and well-known protocols or ontologies [54], or simply when the opening of the data requires proprietary software [54]. Research data is available in varying formats [55] and the lack of harmonization of data formats, processing, analyses and data transfers [18] altogether inhibits open data use. Other inhibitors include the data’s very nature (i.e. some are more easily reused than others) [40], the multiplicity of data types [13], the lack of a clear data usage license [54], the large volume and size of the data [48], the lack of awareness regarding existing standards for data citation [13], along with an access fee needed to access such data [55].

## Discussion

In the previous section, researchers’ drivers and inhibitors for openly sharing and using research data were examined, as derived from the selected studies. The identified factors were detailed in each of the eleven factor categories. In this section, both the findings and their implications are discussed.

### Open research data theory development

The results section shows that of the 32 selected studies, nine mention theories. Few theories have been used or applied, and even fewer have been extended or developed. This finding confirms the study by Kim and Adler [42] that had a similar finding specifically for studies concerning sharing data openly. In this section, the potential for theory development in research concerning open research data sharing and use is discussed.

There might be multiple possible explanations for the limited use, application, development and testing of theories in open research data research. First, researchers whose research interests concern research data might not be aware of potential existing theories for open data research. This might have to do with the fact that there is no such thing as an open research data theory. Open research data is multifaceted, as explained in previous sections that indicate that different theories with different foci are required. Theories from related research disciplines, such as public administration, information systems, and psychology altogether do provide many theories that contain some constructs similar or related to the categories and factors derived from our thematic analysis (Table 8). Such theories can be used as bases for building that extending or further developing an open research data theory. For example, the “New Institutional Theory” [61, 62] refers to regulative pressures, and the “Cognitive Evaluation Theory” [63, 64] refers to intrinsic motivations. Different elements of various existing theories might be combined to create a more comprehensive theory that can be used to better understand, explain and address possible challenges related to both open research data sharing and use.

**Table 8:** Overview of theories (examples) related to factors identified through our thematic analysis that might potentially be used for open research data theory development.

	*Categories derived from our thematic analysis*	Examples of identified factors included in existing theories	Examples of theories (partly) addressing the identified factors
*1*	*Background*	Age and gender	The extended Unified Theory of Acceptance and Use of Technology (UTAUT2) [65]
*2*	*Requirements and formal obligations*	Voluntariness of use	Unified Theory of Acceptance and Use of Technology (UTAUT) [66]
*3*	*Personal driver / Intrinsic motivations*	Intrinsic motivation	Cognitive Evaluation Theory [63, 64]
*4*	*Facilitating conditions*	Curiosity and joy	Hedonic-Motivation System Adoption Model (HMSAM)) [67]
		facilitating conditions	The integrated UTAUT-ECT (Expectation Confirmation Theory) Theory of Information Systems continuance [68]
*5*	*Trust*	Trust	UTAUT-ECT Theory of Information Systems continuance [68]
*6*	*Expected performance*	Performance expectancy	The extended Unified Theory of Acceptance and Use of Technology (UTAUT2) [65]
		Reputation and sense of achievement	Equity Theory [69–71]
		Rewards	Two Factor Theory of motivation [72–75], Expectancy Theory [76]
*7*	*Social influence and affiliation*	Support of colleagues	Equity Theory [69–71]
		Norms of the social system	Innovation Diffusion Theory [77]
*8*	*Effort*	Skills, time and education	Equity theory [69–71]
*9*	*Experience and skills*	Experience	ARCS Motivational Model [78, 79]
*10*	*Legislation and regulation*	Regulative pressures	New institutional theory [61, 62]
*11*	*Data characteristics*	Ease of use	Multi-motive Information Systems Continuance model [80]

Another possible explanation for the limited mention, use, application and development of a theory in the studies selected for the literature review is that open data researchers might have found that existing theories are not useful for examining open research data sharing and use. None of the theories listed in Table 8 are readily fit to address the challenges surrounding open research data sharing and use. Thus, this calls for the development of a new theory, for which the categories and factors derived from our thematic analysis can be used as a basis. And such theory should build on the existing theories by altogether integrating, testing and complementing them.

### Potential application of categories and factors

This study’s overview of categories and factors can be used in future research concerning drivers and inhibitors for open research data sharing and use. Also, this overview can provide insights and guidance to other stakeholders at the institutional level and for national funders’ open science policies. This potential is discussed in the following subsections:

#### Potential for related research fields

This study conducted a thorough, comprehensive systematic literature review that collects metadata and facts from 32 prior open research data studies. Per the systematic literature review results, developed was an overview of categories and factors influencing open research data adoption to facilitate researchers in the related fields to comprehend various factors, including: individual considerations such as trust and perceived effort; a researcher’s context; and many other motivation factors, such as discipline practices and expectations. The literature review shows that the overview of categories and factors provides a more holistic explanation of why researchers are driven or inhibited to share and use open research data than existing research has done so far. In the future, the overview can be used to further examine researchers’ drivers and inhibitors for both sharing and using open data in different research disciplines and contexts, such as disciplines with low rates of data sharing and use versus disciplines with higher rates of data sharing and reuse. With the factor overview as a starting point, researchers can investigate under which conditions different types of researchers (from different research disciplines, functioning in different institutional contexts) can be both stimulated and incentivized to share and use open research data. This is vital to realize the envisioned benefits of both sharing and using open research data and finally generate both newer insights and advance scientific knowledge.

#### Developers of open research data infrastructures

Developers of open research data infrastructures need to take the factors underlying the factor overview into account as the needs of individual researchers can be derived from them. For example, “lack of large data repositories” inhibitor indicates to developers that such repositories might need to be developed. Infrastructure developers can thus further examine which drivers and inhibitors should be prioritized according to researchers in different research disciplines, countries and positions. And developers can use the factor overview to develop infrastructures that support both open research data sharing and use.

#### Professional librarians

The derived overview of categories and factors influencing open research data adoption can assist institutions that need to both serve and support the researchers working in such institutions. The eleven categories and factors altogether underlying the overview can be the first step for academic libraries and other research support organizations (e.g., the office of research or grant management services) to develop effective data services, workflows and consultations for their researchers. As both specifically and practically, survey instruments can be developed, and that the researchers’ maturity levels on open data sharing and reuse can be measured per both Fig 2 (the macro level with categories) and Table 8 (the micro level with specific factors).

#### Open data and open science policy makers, advisors and funding bodies

Finally, both the overview of categories and factors impacting open research data adoption can serve as strong references for open data and open science policy makers, advisors, and funding bodies altogether to recognize both the drivers and inhibitors of researchers’ open data sharing and use practices. The factor overview is the first vital step that allows them to create strategies that incentivize both open research data sharing and use. The incentive mechanisms should incorporate the factors included in such overview.

## Conclusions

This study’s purpose is to systematically review the literature on individual researchers’ drivers and inhibitors for both sharing and using open research data. With a “Systematic Literature Review” approach complemented with a snowballing approach, 32 studies describing research into open data sharing and use were selected. All studies were published between 2004 and 2019 inclusively. Nearly half of the selected studies (n = 15) is conducted by quantitative approaches; twelve are qualitative, and five use a mixed-method approach. Most studies (n = 22) focus on a specific research discipline, such as biodiversity, social sciences, or microarray science. The majority of such as investigated studies (n = 18) do not mention any theory. Of the fourteen studies that do mention theory, eleven use theory to altogether develop the theoretical research framework or model and/or to test hypotheses. Theories that are mentioned more than once are the “Theory of Planned Behavior” (n = 7), “Institutional Theory” (n = 2), “Technology Adoption Model” (n = 2), integrated “Unified Theory of Acceptance and Use of Technology”, and the two-stage expectation confirmation theory of “Information Systems” continuance (n = 2).

From the identified studies, we synthesized a comprehensive list of: (1) factors driving researchers to openly share research data; (2) factors inhibiting researchers to openly share research data; (3) factors driving researchers to use open research data; (4) factors inhibiting researchers to use open research data. Altogether influencing factors were identified in eleven categories: “the researcher’s background”, “requirements and formal obligations”, “personal drivers and intrinsic motivations”, “facilitating conditions”, “trust”, “expected performance”, “social influence and affiliation”, “effort”, “the researcher’s experience and skills”, “legislation and regulation”, and “data characteristics”. Also found were that the factors impacting both open data sharing and open data use are often similar (e.g. “the researcher’s background” category) that show the strong interdependency between such two activities.

Most drivers for openly sharing research data are related to personal and intrinsic motivations, to the expected performance of researchers and to the effort of openly sharing research data. The identified inhibitors for open data sharing mostly relate to legislation and regulation, facilitating conditions, and expected performance, in the sense that openly sharing research data can lead to worse performance. Drivers for open research data use mainly relate to personal and intrinsic motivations and the expected performance of researchers. The identified inhibitors for open research data use mainly relate to effort and data characteristics. Yet, the number of identified drivers and inhibitors for research data sharing and use does not indicate the importance of these drivers and inhibitors, and further research is needed to examine if certain drivers and inhibitors, in specific contexts and research disciplines, are more important than others.

The large diversity of factors influencing open research data sharing and use shows that theory regarding this topic needs to combine insights from various fields. In the discussion section, we highlighted various theories from information science literature, information systems literature, and motivational psychology literature that might be combined to further develop theory in research into both open research data sharing and use. This study’s analysis of theory development with regard to open research data could thus inspire other researchers while studying specific aspects of open research data sharing and use.

This study contributes to filling the gap of theory development in open data literature by providing a coherent and comprehensive overview of categories and underlying factors that need to be considered when studying open research data sharing and use behavior. With a scattered body of knowledge, this study developed an argument about how the categories and factors are connected to provide the basis for a comprehensive overview of factors influencing open research data adoption. The developed overview is needed to further examine the importance of researchers’ drivers and inhibitors for research data sharing and use in different research disciplines and contexts, such as disciplines with low rates of data sharing and use versus disciplines of high rates of data sharing and reuse. Moreover, while the majority of the inhibitors for open research data sharing and use cannot be mitigated completely, the negative impact of many challenges may be reduced with the right infrastructure and related institutional arrangements. With all these in mind, this study is the first essential step towards designing infrastructures and institutional arrangements that stimulate and incentivize open research data sharing and use behavior, since these need to take into account the factors driving and inhibiting researchers to adopt open research data.

Systematic literature reviews potentially have a risk of bias both at the review level (i.e. analysis of studies) and at the outcome level (i.e. reporting bias). Also, especially in the systematic review of qualitative research, a more robust study quality assessment premise continues to be a challenge [28]. Although these risks and challenges cannot be removed completely, various measures were taken to reduce bias as much as possible. For example, multiple assessors were used for each study included in our review and detailed information was provided about how we collected, assessed and analyzed the collected studies. Thus, by providing transparency by the study’s review protocol and by openly sharing the research data underlying our analysis and findings, other scholars are enabled to cross-check our findings and examine if other interpretations could be possible.

In addition, some of the identified factors driving or inhibiting the adoption of open research data have only been found in a single study. Thus, more evidence is needed to improve our understanding of these factors and to investigate whether they play a role in different contexts. Future research is recommended to empirically test the usability and completeness of the aforesaid factor overview and to adapt it to specific contexts of open data sharing and use behavior. Especially as future research should focus on whether the factor overview needs to be adapted for research data provision and use in specific research disciplines (e.g. astrophysics, genomics, humanities, social sciences, computer science). Furthermore, it should be investigated whether certain factors receive a higher weight in researchers’ trade-off to openly share research data or not, and in their trade-off to use open research data or not. Moreover, most of the studies examined were focused on research data sharing and use in the United States and in European countries, and to a much smaller extent on Asian, African, and other jurisdictions, while the latter should receive more attention. Finally, future research should focus on both designing infrastructures and institutional arrangements that altogether stimulate and incentivize both open research data sharing and use behavior.

## Supporting information

S1 Table Overview of studies included in our literature review. (DOCX)

S2 Table Overview of drivers for openly sharing research data by researchers, identified in the studies included in our literature review. (DOCX)

S3 Table Overview of inhibitors for openly sharing research data by researchers, identified in the studies included in our literature review. (DOCX)

S4 Table Overview of drivers for using open research data by researchers, identified in the studies included in our literature review. (DOCX)

S5 Table Overview of inhibitors for using open research data by researchers, identified in the studies included in our systematic literature review. (DOCX)

S1 Checklist PRISMA 2009 checklist. (DOC)

S1 Fig (DOC)

S1 File (DOCX)

S1 Data (TXT)
