# Supplementary material for: What drives and inhibits researchers to share and use open research data? A systematic literature review to analyze factors influencing open research data adoption
Source: PLoS One. 2020 Sep 18;15(9):e0239283. doi: 10.1371/journal.pone.0239283 (PMC7500699; doi:10.1371/journal.pone.0239283)
Supplement: S1 Table — (DOCX) [file pone.0239283.s001.docx]

**S1 Table. Overview of studies included in our literature review.**

| **No*.*** | **Authors and year of publication** | **Title** | **Study objective** | **Journal (J) / dissertation (D)** |
| --- | --- | --- | --- | --- |
| *1* | Arza and Fressoli [4] | Systematizing benefits of open science practices | This paper aims to disentangle different meanings for open science and organize them so as to relate them with claims on benefits as referenced in the literature. | Information Services & Use (J) |
| *2* | Arzberger, Schroeder [50] | Promoting access to public research data for scientific, economic, and social development | The goals of this article and its recommendations are to ensure that both researchers and the public receive optimum returns on public investments in research, and to build on the value chain of investments in research and its data resource. | Data Science Journal (J) |
| *3* | Bezuidenhout [51] | Technology Transfer and True Transformation: Implications for Open Data | This paper examines research settings in low/middle-income countries (LMIC) to better ­understand ­how ­resource ­limitations influence ­open data ­related activities. | Data Science Journal (J) |
| *4* | Campbell [2] | Access to scientific data in the 21st century: Rationale and illustrative usage rights review | This paper reviews some of the forces pushing for more open access to scientific data in the 21st century. | Data Science Journal (J) |
| *5* | da Costa and Leite [47] | Factors influencing research data communication on Zika virus: a grounded theory | This paper aims to report on investigation results, and to propose a theoretical model to represent factors influencing research data communication about the Zika virus from the perspective of biological science and of health science researchers | Journal of Documentation (J) |
| *6* | Cragin, Palmer [52] | Data sharing, small science and institutional repositories | This study intends to inform institutional repository development. | Philosophical Transactions of the Royal Society A: Mathematical, Physical and Engineering Sciences (J) |
| *7* | Curty, Crowston [40] | Attitudes and norms affecting scientists’ data reuse | This study uses the Theory of Reasoned Action to test the relationship between the beliefs and attitudes of scientists regarding data reuse, and their self-reported data reuse behavior. | PLOS ONE (J) |
| *8* | Enke, Thessen [10] | The user's view on biodiversity data sharing — Investigating facts of acceptance and requirements to realize a sustainable use of research data | This study investigates a) motivational and social aspects for reluctant data sharing, b) data handling strategies currently in use and c) technical requirements to identify prerequisites for optimized data sustainability. | Ecological Informatics (J) |
| *9* | Fecher, Friesike [11] | What drives academic data sharing? | This study aims to develop a conceptual framework that explains the process of data sharing from the primary researcher’s point of view. | PLOS ONE (J) |
| *10* | Ganzevoort, van den Born [53] | Sharing biodiversity data: citizen scientists’ concerns and motivations | This study's objective is to gain insight into the interrelated factors of volunteer biodiversity recorders’ backgrounds, motivations, and views on sharing data, in order to learn more about what drives citizen scientists to record biodiversity, and to assist practitioners in connecting with their volunteers’ concerns and motivations. | Biodiversity and Conservation (J) |
| *11* | Grechkin, Poon [6] | Wide-Open: Accelerating public data release by automating detection of overdue datasets | This paper aims to describe the developed Wide-Open, a general approach that applies text mining to automatically detect overdue datasets in a public repository. | PLOS biology (J) |
| *12* | Haeusermann, Greshake [18] | Open sharing of genomic data: Who does it and why? | The objective of this study is to explore the characteristics and motivations of people who, having obtained their genetic or genomic data from Direct-To-Consumer genetic testing (DTC-GT) companies, voluntarily decide to share them on the publicly-accessible web platform openSNP. | PLOS ONE (J) |
| *13* | Harper and Kim [41] | Attitudinal, normative, and resource factors affecting psychologists’ intentions to adopt an open data badge: An empirical analysis | The purpose of this study is to investigate the attitudinal, normative, and resource factors affecting psychologists’ adoption of an open data badge. | International Journal of Information Management (J) |
| *14* | Joo, Kim [17] | An exploratory study of health scientists’ data reuse behaviors: Examining attitudinal, social, and resource factors | The purpose of this paper is to examine how health scientists’ attitudinal, social, and resource factors affect their data reuse behaviors. | Aslib Journal of Information Management (J) |
| *15* | Kim and Adler [42] | Social scientists’ data sharing behaviors: Investigating the roles of individual motivations, institutional pressures, and data repositories | The purpose of this study is to locate individual, institutional, and resource factors that influence data sharing behaviors among social scientists. | International Journal of Information Management (J) |
| *16* | Kim and Yoon [43] | Scientists’ Data Reuse Behaviors: A Multi-Level Analysis | This study explores the factors that influence the data reuse behaviors of scientists and identifies the generalized patterns that occur in data reuse across various disciplines. | Journal of the Association for Information Science and Technology (J) |
| *17* | Mooney and Newton [13] | The anatomy of a data citation: Discovery, reuse, and credit | This study examines author behavior and sources of instruction in disciplinary and cultural norms for writing style and citation via a content analysis of journal articles, author instructions, style manuals, and data publishers. | Journal of Librarianship and Scholarly Communication (J) |
| *18* | Piwowar and Vision [9] | Data reuse and the open data citation advantage | This study aims to look at citation rates while controlling for many known citation predictors and investigate the variability of data reuse. | PeerJ (J) |
| *19* | Piwowar, Day [8] | Sharing detailed research data is associated with increased citation rate | The purpose of this study is to examine the citation history of 85 cancer microarray clinical trial publications with respect to the availability of their data. | PLOS ONE (J) |
| *20* | Raffaghelli and Manca [54] | Is there a social life in open data? The case of open data practices in educational technology research | The aim of the study is to show how open data related practices are emerging, and to which extent these align with the principles of open science. | Publications (J) |
| *21* | Sá and Grieco [1] | Open data for science, policy, and the public good | This article examines the unlikely case of Brazil’s National Institute for Space Research’s transition toward an open data model. This study then addresses the question: how and why did INPE develop its open data model? | Review of Policy Research (J) |
| *22* | Sayogo and Pardo [49] | Exploring the determinants of scientific data sharing: Understanding the motivation to publish research data | This study was designed to contribute greater understanding of the behavior in publishing research data by correlating the challenges to the propensity of researchers to openly share their data. Using the survey response from DataONE, this paper will address two main research questions: 1) what are the critical challenges facing individual researchers in publishing their research data openly to the public, and 2) to what extent do these challenges influence the propensity of researchers to openly share their data sets? | Government Information Quarterly (J) |
| *23* | Schmidt, Gemeinholzer [55] | Open Data in Global Environmental Research: The Belmont Forum’s Open Data Survey | This paper presents the findings of the Belmont Forum’s survey on open data which targeted the global environmental research and data infrastructure community. | PLOS ONE (J) |
| *24* | Tenopir, Allard [56] | Data Sharing by Scientists: Practices and Perceptions | This article reports the results of a survey of scientists' current data sharing practices and their perceptions of the barriers and enablers of data sharing. | PLOS ONE (J) |
| *25* | Wallis, Rolando [57] | If we share data, will anyone use them? Data sharing and reuse in the long tail of science and technology | In this article, we explore data sharing practices among scientists and technology researchers in a National Science Foundation Science and Technology Center. | PLOS ONE (J) |
| *26* | Yoon [58] | Data reusers' trust development | The objective of thist study is to explore data reusers’ processes of making trust judgments about data. | Journal of the Association for Information Science and Technology (J) |
| *27* | Yoon and Kim [44] | Social scientists’ data reuse behaviors: Exploring the roles of attitudinal beliefs, attitudes, norms, and data repositories | This study explores the data reuse behaviors of social scientists in order to better understand both the factors that influence those social scientists' intentions to reuse data and the extent to which those factors influence actual data reuse. | Library and Information Science Research (J) |
| *28* | Zenk-Möltgen, Akdeniz [45] | Factors influencing the data sharing behavior of researchers in sociology and political science | The purpose of this paper is to investigate how different institutional and individual factors affect the data sharing behavior of authorsof research articles in sociology and political science. | Journal of Documentation (J) |
| *29* | Zimmerman [59] | Not by metadata alone: The use of diverse forms of knowledge to locate data for reuse | This study aims to examine the processes by which ecologists locate data that were initially collected by others. | International Journal on Digital Libraries (J) |
| *30* | Zuiderwijk [19] | Open data infrastructures: The design of an infrastructure to enhance the coordination of open data use | The objective of this study is to develop an infrastructure that enhances the coordination of open government data use by researchers. | Publisher BOXPress (D) |
| *31* | Zuiderwijk and Cligge [46] | The acceptance and use of open data infrastructures-drawing upon UTAUT and ECT | This study aims to examine to which extent and by which factors the acceptance and use of open data infrastructures by researchers can be influenced. | Conference on Electronic Government and Electronic Participation (EGOV) |
| *32* | Zuiderwijk and Spiers [48] | Sharing and re-using open data: A case study of motivations in astrophysics | This study seeks to provide in-depth insight about the complex interaction of factors influencing motivations for sharing and re-using open research data within a single discipline, namely astrophysics. | International Journal of Information Management (J) |
