# Supplementary material for: What drives and inhibits researchers to share and use open research data? A systematic literature review to analyze factors influencing open research data adoption
Source: PLoS One. 2020 Sep 18;15(9):e0239283. doi: 10.1371/journal.pone.0239283 (PMC7500699; doi:10.1371/journal.pone.0239283)
Supplement: S2 Table — (DOCX) [file pone.0239283.s002.docx]

**S2 Table. Overview of drivers for openly sharing research data by researchers, identified in the 32 studies included in our literature review.**

| ***No.*** | **Source** | **Factors driving researchers to openly share research data** |
| --- | --- | --- |
| *1* | Arza and Fressoli [4] | Equal access to publicly funded data; Open up opportunities to participate in new international projects widening local scientists’ networks; Social responsiveness; Increase scientific efficiency; Through interaction with other actors, research agenda's could be better guided towards solving problems affecting a specific group; Finding cheaper solutions to societal problems; Help local problems to become visible and better communicated; Better inform society and foster new processes of learning; Other people can offer inputs for develop final solutions |
| *2* | Arzberger, Schroeder [50] | Appropriately designed technological infrastructure; Broad international agreement on interoperability; Effective data quality controls; A variety of institutional models and tailored data management approaches that meet the needs of researchers; Continued and dedicated budgetary planning and appropriate financial support; National laws and international agreements; Appropriate reward structures; Support from National and local governments (in terms of policies, programs, management practices); Better ICT facilitation (internet hosts per person, percentage of computers per household, Continued rate of growth of chip, storage, and network technology capacity); More data production; Systematic visibility of the data source; Professionalism (build on codes of conduct and ethics of the scientific community); Greater returns of public investment in research; Generate wealth through the downstream commercialization of outputs; Provide decision-makers with the necessary facts to address complex, often trans-national problems; Offer individuals the opportunity to better understand the social and physical world in which we all live; Code of conduct and related normative standards of professional scientists and their communities |
| *3* | Bezuidenhout [51] | None mentioned |
| *4* | Campbell [2] | Effective data preservation and archiving; Encourage validation of research results; Avoidance of duplication; Encourage economic development; Understanding what users may, or may not, do with data in online data repositories; Spur innovation; More evaluation capability (e.g. other researchers testing the data and hypotheses); Good management of data integrity over time; The ability to grow storage and access capabilities and still operate reliably and efficiently |
| *5* | da Costa and Leite [47] | Being acknowledged, open-working academic culture, data involve no human subjects (e.g., patients); data's nature is quantitative; the requirement to make the data available in proper repositories as a condition to publishing papers; research discipline/area (e.g. Biology researchers are more inclined to openly share data than Medicine and Pharmacy); researchers' age (younger researchers are more inclined to openly share their data, both due to their abilities in the use of technologies and to their interest in collaborating with other research projects); the possibility of publishing the research results in journals of great international prestige; adequate funding for the treatment and availability of data can generate savings in resources in future research funding; the hiring of data specialists; appropriate information systems, such as software and equipment that reduces the effort required by researchers in producing and disclosing data; the availability of research data repositories (repositories are expected to reduce the effort required for data registry and, simultaneously, generate more visibility for the research and for the institutions where the research was conducted) |
| *6* | Cragin, Palmer [52] | Data format appropriate for data sharing and re-use; possibility to better advance the area of research; increased willingness to make data available when data were cleaned, processed, refined and analyzed during the research; form of data appropriate for data sharing and re-use; small amount of work and time; short embargo period; re-use value for many years; size of the research community and the extent to which data is viewed as a tremendous asset; trust; the possibility of data management consultation |
| *7* | Curty, Crowston [40] | Personal benefits (e.g. increasing citation rates); Mandates for research data sharing; Compliance with governmental directives; The expectation that data will be re-used; Culture of data sharing |
| *8* | Enke, Thessen [10] | Transparency of study results; High availability of comparable data sets for comprehensive analyses ; Network with other scientists for interdisciplinary studies; As data was generated with public money it should be made public; Researchers' visibility in the community increases |
| *9* | Fecher, Friesike [11] | Recognition for data sharing (career prospects); Character traits (Big Five: openness to experience, conscientiousness, extraversion, agreeableness, neuroticism); Research activity ( Individuals who work solely in research, in contrast to researchers who have time-consuming teaching obligations, are more likely to make their data available to other researchers.); Having a say in the data use; Formal recognition; Professional exchange; Quality improvement; Data sharing policy and organizational culture; Data management; Funding policy (grant requirements); Financial compensation; Disciplinary practice; Formatting standards (impact metrics need to be adapted to foster data sharing); Interoperability; Scientific progress; Exchange; Review; Synergies; Journal policy; Informed consent; Contractual consent; Falsification; Commercial misuse; Competitive misuse; Flawed interpretation; Unclear intent; Data security conditions; Performance; Data storage; Data security, tools and applications; Technical support; Data documentation and metadata; Metadata standards |
| *10* | Ganzevoort, van den Born [53] | Clear and transparent data policy; Easily digestible form |
| *11* | Grechkin, Poon [6] | Creating regular expressions for dataset identifiers; Identifying the web API for dataset access; Adapting the query-result parser to distinguish between invalid UIDs, datasets that have been released, and datasets that remain private; The open-access movement championed by journals |
| *12* | Haeusermann, Greshake [18] | Wanting to learn about themselves; Contributing to the advancement of research; Improving the predictability of genetic testing; Minimal privacy risks; Data generally collected and studied in silos; Take a risk in the privacy and data-protection realm; Fun to explore data |
| *13* | Harper and Kim [41] | Increase in transparency of the research method and process; No data replication required ; Reducing costs; Saving time involved in data collection; Allow researchers to confirm the findings of the original publication or to test different hypotheses; Potential for collaboration among scholars with similar research interests; Ethic codes; Standard social norms; Perceived benefits (institutional recognition, professional recognition, additional citations and other academic rewards); Normative factors (e.g. subjective norm and pressure by journals); Resource factors (e.g. availability of a data repository, knowledge of metadata and its practices, and data sharing requirements of a journal) |
| *14* | Joo, Kim [17] | Allows other researchers to explore new interpretations of data; Data reuse can lead to more findings from the same data set and increase the knowledge in the field; Individual incentives; Infrastructure; Trust; Data discovery; Academic discipline / sector; Accelerated scientific progress; Reduced research costs; Increased visibility and relevance of research output; Educating researchers about the consumer side of open data practices; An initial large data repository to foster data sharing and reuse culture; Regulatory pressure; - Attitudes toward data sharing; The availability of data repositories |
| *15* | Kim and Adler [42] | Perceived career benefit; Attitude toward data sharing; Perceived normative pressure; Institutional pressures by funding agencies and journals (not significant); Data repository (+)(-); Increasing the transparency of quantitative analytic work; Lending more credibility to research findings; Providing evidence to support an analytics framework and decision; A source for researchers to consult when considering how to build upon existing studies; Replication and verification is made immediately possible; Allow testing different hypotheses; Building better research studies; Facilitate participation from multiple perspectives; Allowing access of the data for more disciplines and for researchers from different backgrounds; Reduce costs by avoiding the publication of data collection efforts; Contribute to the education of students; Journal policies; Received funding from government agencies |
| *16* | Kim and Yoon [43] | None mentioned |
| *17* | Mooney and Newton [13] | Reward structures; Increased data citation; Providing repositories and archives |
| *18* | Piwowar and Vision [9] | Increased number of citations; Allow valuable resources to contribute far beyond their original analysis; Help to identify errors; Discourage research fraud; Useful for training new researchers; Increase efficient use of funding and population resources by avoiding duplicate data collection; Increased data use; Facilitated credibility determination; Improved discoverability; A focus on best work through data availability |
| *19* | Piwowar, Day [8] | Increased number of citations; Encourage multiple perspectives; Help to identify errors; Discourage fraud; Useful for training new researchers; Increase efficient use of funding and population resources by avoiding duplicate data collection; Peer pressure to share data |
| *20* | Raffaghelli and Manca [54] | None mentioned |
| *21* | Sá and Grieco [1] | Efficient use of resources; Ensure the validity of the data by multiple users; Enable citizen science; Encourage public activism |
| *22* | Sayogo and Pardo [49] | Data management skills; Organizational support for data management; The acknowledgement of the dataset's originator in terms of appreciation(e.g. co-authorship on publications, formal acknowledgement of the data providers, opportunity to collaborate with others); Legal and policy requirements (e.g. significance of citation, legal agreement, statement of use, conditions of use, and approval for reuse) |
| *23* | Schmidt, Gemeinholzer [55] | Sense of responsibility about acceleration of scientific research; Sense of responsibility about dissemination and recognition of research results; personal commitment to open data and respond requests from data users; funder policies; publisher policies |
| *24* | Tenopir, Allard [56] | Verification of results; Extending research from prior results; Formal citation; Sharing reprints; Mandates for data management plans from federal agencies; World-wide attention to the need to share and preserve data; Re-analysis of data helps verify results data; Different interpretations or approaches to existing data contribute to scientific; Well-managed, long-term preservation helps retain data integrity; When data is available, (re-)collection of data is minimized; thus, use of resources is optimized; Data availability provides safeguards against misconduct related to data fabrication and falsification; Replication studies serve as training tools for new generations of researchers; Discipline-related culture of data sharing (e.g. more sharing in geophysics, biodiversity, and astronomy); Formal organizational policy; Policies and assistance with data management across the data lifecycle; The ability to place conditions on data access; Receiving proper data citation credit; The ability to review works derived from the dataset; Age; older people (over 50) show more interest in sharing data; Geographic location |
| *25* | Wallis, Rolando [57] | Big science (that has richer investment of funding, labors, scale, and infrastructure); Under increased pressure to release data; Need to avoid repeating the work of the past by working together and sharing data; Positively affect the progress of science; Enable other researchers to ask new questions; Recognized that others may find the data interesting or useful; Make results of publicly funded research available to the public; Facilitating other researchers' ability to pursue new lines of research; Demonstrating the value of their own accomplishments; Facilitating comparisons between methods and sites; Promulgating their technology as a basis for others' research) |
| *26* | Yoon [58] | None mentioned |
| *27* | Yoon and Kim [44] | None mentioned |
| *28* | Zenk-Möltgen, Akdeniz [45] | Past data sharing; research discipline (e.g. more data sharing in political science than in sociology), positive attitude towards data sharing; perceived social pressure to share data with others; perceived behavioral autonomy and self-efficacy to be able to share data |
| *29* | Zimmerman [59] | To share portions of a dataset rather than to share the whole dataset; using measures that make the collection and interpretation of the data easier |
| *30* | Zuiderwijk [19] | Gaining new insight for data-driven research; Generation of new datasets, information, and knowledge when data from various sources are combined; Permitting in depth public scrutiny by making it easier to analyze, process and combine data |
| *31* | Zuiderwijk and Cligge [46] | None mentioned |
| *32* | Zuiderwijk and Spiers [48] | Researcher’s background; personal drivers; experience; legislation; regulation and policy; data characteristics; performance expectancy, usability, collaboration; raw data is infrequently collected directly by the researchers;  exterior public data is shared automatically; supportive data sharing culture; lack of concerns about ethics and commercial potential of data; effort: preventing duplication of effort so that less time and resources are wasted; Expected performance; Visibility: research work done gets more attention after it has been shared openly, noticed for example by an increased number of citations and an increased profile; trust: reproducibility of results and the fact that anyone can access the data, improve the quality of the research; Personal drivers / intrinsic motivations: Better science, move the field forward more quickly and easily |
