# Supplementary material for: What drives and inhibits researchers to share and use open research data? A systematic literature review to analyze factors influencing open research data adoption
Source: PLoS One. 2020 Sep 18;15(9):e0239283. doi: 10.1371/journal.pone.0239283 (PMC7500699; doi:10.1371/journal.pone.0239283)
Supplement: S3 Table — (DOCX) [file pone.0239283.s003.docx]

**S3 Table. Overview of inhibitors for openly sharing research data by researchers, identified in the 32 studies included in our literature review.**

| ***No.*** | **Source** | **Factors inhibiting researchers to openly share research data** |
| --- | --- | --- |
| *1* | Arza and Fressoli [4] | None mentioned |
| *2* | Arzberger, Schroeder [50] | National security; Privacy and the protection of trade secrets; Authorship; Producer credits; Ownership; Financial arrangements (and budgets); Licensing terms; Where appropriate, restrictions on use for private intellectual property rights; Technological issues |
| *3* | Bezuidenhout [51] | Concerns about the speed of data analysis; differences in available resources (equipment) which slows down the pace of research and which makes it even more important to only release data once the related publication is out (the specter of “being scooped” due to the slower pace of research); concerns that if data would be released it would not be re-used by international peers because of anxiety linked to the equipment used to produce it; the helplessness of changing the pace at which data are generated; the limited availability of technologies that underpin data engagement activities (e.g. lack of ICTs for dissemination, lack of platforms; lack of appropriate software); Lack of expertise, older equipment, poor maintenance and technical support and infrastructural challenges (such as power provision); bureaucracy for research funding reimbursement; the limited availability of technologies that underpin data engagement activities (e.g. lack of ICTs for re-use, lack of online platforms, lack of appropriate software, lack of analysis procedures, lack of ICTs for curation and storage; lack of analysis software); fear of few re-use |
| *4* | Campbell [2] | Unclear what ‘openness’ means (large variety of licenses); Legal rights and restrictions) |
| *5* | da Costa and Leite [47] | Data sensitivity (e.g., no distribution to patient data); data's nature is qualitative; The communication of research data does not receive as much academic prestige as papers; lack of recognition of the citation of the research data as compensatory for the effort involved in collecting the data for researchers; lack of information systems to disclose raw research data in certain research disciplines (e.g. medicine); privacy issues; data processing requiring more time and effort (as it is necessary to structure the data according to a given standard, or describe the data more thoroughly than the original research would require) |
| *6* | Cragin, Palmer [52] | Data format not appropriate for data sharing and re-use; form of data inappropriate for data sharing and re-use; large amount of work and time; short embargo period; short re-use value; dataset too large to share; lack of trust; concerns about data misuse; the desire for personal control of one’s research products; the lack of proper recognition or reward; data misuse incidents (disregard of good faith practices); concern about wrong or inappropriate interpretation of data; improper citation of data; scientists' reputation at risk |
| *7* | Curty, Crowston [40] | None mentioned |
| *8* | Enke, Thessen [10] | Loss of control; Time investment (the amount of time they would have to invest to get the data ready to share); Fear of potential violation of property rights (intellectual property or patent issues); Confidentiality or legal issues; No acknowledgement for my effort; Quality of my data; Lack of data standards; Someone draws wrong conclusions; Criticism on my data or analyses- Fear of potential violation of property rights; Fear of loss of data autonomy (e.g., control over unpublished data in publicly accessible online database); Technology-related limitation (e.g., reluctance to use online databases because of complex user inter- faces making data entry time consuming) |
| *9* | Fecher, Friesike [11] | Character traits (Big Five: openness to experience, conscientiousness, extraversion, agreeableness, neuroticism); Research activity ( Individuals who work solely in research, in contrast to researchers who have time-consuming teaching obligations, are more likely to make their data available to other researchers.); Seniority in the academic system (non-tenured researchers are less likely to share their research data openly); Nationality in relation to national research policies (e.g. German and Canadian scientists are more reluctant to share research data publicly than their US colleagues); Knowledge about data requester ( To the question why one would not share research data: “I have doubts about others being able to use my work without control from my side” ); Potential harm; Ownership and right of use; Privacy; Copyright; Priority rights for publications; Time and effort; Skills and knowledge (Missing knowledge further relates to poor curation and storing skills); Financial resources; Confidentiality; Commercialization of research findings; Data quality |
| *10* | Ganzevoort, van den Born [53] | Trust; Ownership; Collected biodiversity data be used for the ‘wrong’ reasons |
| *11* | Grechkin, Poon [6] | Required manual efforts |
| *12* | Haeusermann, Greshake [18] | Aware of the privacy risks of their involvement in open genetic data sharing |
| *13* | Harper and Kim [41] | Perceived risks (missing out on future publication opportunities, misuse of researcher’s data and privacy-related concerns; Perceived effort (when something takes too much effort or there is not enough time or funding to organize the data researchers are less likely to engage in data sharing behaviors) |
| *14* | Joo, Kim [17] | Concerns about data integrity, technical challenges, privacy and confidentiality, and intellectual property; Data sensitivity; Concern about losing an advantage in their research area |
| *15* | Kim and Adler [42] | Perceived career risk; Perceived effort; Losing commercialization opportunities; Privacy issues; Contracts with industry sponsors; Data repository (+)(-) (the collection of qualitative data – focus groups, interviews, and ethnographic research (i.e., observations) now involves confidentiality agreements that would make data sharing difficult or impossible.”) |
| *16* | Kim and Yoon [43] | None mentioned |
| *17* | Mooney and Newton [13] | Fear of receiving no credit ; Losing funding or publishing opportunities; Someone else publishing with no reward given to the sharer since there is no system of acknowledgement; Intellectual property rights; References to the name of the data creators and publishers are scarce or not prominently featured (mostly references to the dataset title) |
| *18* | Piwowar and Vision [9] | Data must be documented, formatted, and uploaded; Investigators may be afraid that other researchers will find errors in their results; Fear of results scooping additional analyses researchers have planned for the future |
| *19* | Piwowar, Day [8] | The data have to be formatted, documented, and released, which takes considerable time investment; Decide where to best publish data, since supplementary information and laboratory sites are transient; Fear that the original conclusions may be challenged by a re-analysis, whether due to possible errors in the original study, a misunderstanding or misinterpretation of the data, or simply more refined analysis methods; Fear that additional relationships will be discovered in the data; Fear that researchers will be deluged with requests for assistance, or need to spend time reviewing and possibly rebutting future re-analyses; Decrease of their own competitive advantage, whether future publishing opportunities, information trade-in-kind offers with other labs, or potentially profit-making intellectual property; Complicated to release data ; Informed consent agreements may not obviously cover subsequent uses of data and de-identification can be complex; Study sponsors, particularly from industry, may not agree to release raw detailed information; Data sources may be copyrighted such that the data subsets cannot be freely shared |
| *20* | Raffaghelli and Manca [54] | Lack of tools to observe data metrics |
| *21* | Sá and Grieco [1] | Legal implications: public access may negatively impact national security; Lack of control of the scientific findings and conclusions derived from the data; Financial barriers: loss of potential licensing revenue that would accrue to inventors of patentable discoveries; Organizational: institutional members sometimes resist change; Operational: conveying information to the public is not always straightforward; Openness of ICT tools which helps in opening the data; National security (Depending on the sensitivity of the information, public access may negatively impact national security); Communication of the open data results; Quality of the open data platforms and credibility |
| *22* | Sayogo and Pardo [49] | Issues of data quality, standards, and protection; Issues of ethical responsible use of shared data; Technological barriers: data architecture (data quality and standardization) and data protection (data misuse or scooping); Social, organizational, and economical barriers: 1) cost of sharing (e.g. time and effort), 2) incentives and merit system (lack of sufficient rewards and incentives for researchers), 3) the culture of open sharing (promotion for academe is tied to publication and not data), and 4) structural conflicts and managerial practices in organization (e.g. security reasons, financial interest); Legal and policy barriers (e.g. the lack of rigidity of policies); Local contexts and specificity (e.g. the complexity of the data): specificity of purpose, specificity of events, specificity of methodology, and the duration of re-search; Age: younger researchers tend to share more; Gender: the probability of not publishing data sets is higher than the probability of publishing some, most, or all of the data sets for male respondents |
| *23* | Schmidt, Gemeinholzer [55] | Considering licenses a burden, difficulties in understanding licenses, lack of standardization, concerns about too restrictive licenses (in particular Non Commercial, Share Alike), concerns about misuse of data, attribution stacking (in relation to CC-BY) and issues of confidentiality (i.e. providing levels of open access); intellectual property issues; the desire to publish results before releasing data; legal constraints; loss of credit or recognition and possible misinterpretation or misuse; concerns about legal liability for data or release of data; size of data, laziness, lack of funder requirements to publish data but also if too many data policy apply; lack of attribution and credit; difficulty of using standards; the amount of time or costs that it takes to properly document the data so that it is useful for others; age (respondents of age 20–35 also expressed significantly higher concerns about the impact of data release compared to respondents of age 51 and older.) |
| *24* | Tenopir, Allard [56] | Insufficient time ; Lack of funding; Many organizations do not provide support to their researchers for data management both in the short and long-term; Formal organizational policy; Lack of organizational support for data management; Lack of training of best practices for data management; lack of access to data generated by other researcher or institution; Data may be misinterpreted due to complexity of the data; Data may be misinterpreted due to poor quality of data; Data may be used in other ways than intended; Having no place to put the data; Lack of standards; Sponsor does not require data sharing; Discipline-related culture of data sharing (e.g. less sharing in medical fields and social sciences); Age; younger people are less likely to make their data available to others; Geographic location |
| *25* | Wallis, Rolando [57] | Small science (that has less investment funding, labors, scale, and infrastructure); making data from the long tail discoverable and reusable is emerging as a major challenge; Lack of appropriate infrastructure; Concerns about protecting the researcher's right to publish their results first; Incentive systems that favor publishing articles over publishing data; Difficulty in establishing trust in others' data; Individual investment needed to preserve and manage data; "Gift culture" of scholarship (i.e., Researchers exchange valuable data through only trusted relationships, not for the public); Researchers may lose the ability to barter data privately, thus creating a disincentive for deposit; The mobility of data (i.e., data is hard to be moved to other facilities); Metadata is not always consistent |
| *26* | Yoon [58] | None mentioned |
| *27* | Yoon and Kim [44] | None mentioned |
| *28* | Zenk-Möltgen, Akdeniz [45] | Negative attitude towards data sharing |
| *29* | Zimmerman [59] | Issues of ownership; lack of reward for sharing data |
| *30* | Zuiderwijk [19] | Institutional and legal barriers; Data sensitivity; Privacy concerns; Fear to harm the reputation of politicians and organizations that publish the data; For certain types of data the law prohibits their publication; Datasets created by multiple organizations which have different levels of security, different policies and which have to comply with different laws; all need to give permission for the disclosure of the data; Biased data or data of low quality; Fear of the misinterpretation and misuse of open data |
| *31* | Zuiderwijk and Cligge [46] | None mentioned |
| *32* | Zuiderwijk and Spiers [48] | Expected performance, social influence / affiliation: competition and the fear of being scooped; facilitating conditions: lack of time, authorship issues and getting permission from all partners in large collaborations; facilitating conditions and experience: lack of facilitating platforms; data characteristics: limited data usability, the large volume and size of the datasets; personal drivers / intrinsic motivations: the fear that others will find mistakes |
