# Supplementary material for: What drives and inhibits researchers to share and use open research data? A systematic literature review to analyze factors influencing open research data adoption
Source: PLoS One. 2020 Sep 18;15(9):e0239283. doi: 10.1371/journal.pone.0239283 (PMC7500699; doi:10.1371/journal.pone.0239283)
Supplement: S4 Table — (DOCX) [file pone.0239283.s004.docx]

**S4 Table. Overview of drivers for using open research data by researchers, identified in the 32 studies included in our literature review.**

| ***No.*** | **Source** | **Factors driving researchers to use open research data** |
| --- | --- | --- |
| *1* | Arza and Fressoli [4] | Arrive to new findings; Reproducibility of key research findings (and also experimental methods) that could push science ahead; Digital tools (e.g. the possibility to involve more actors in data collection through citizen science platforms, not restricted by physical or cognitive distance); Provides a democratic scientific knowledge sharing platform : "Open access increases the pool of information available to anyone not just scientists"; Allows collaboration across diverse groups |
| *2* | Arzberger, Schroeder [50] | Reinforces open scientific inquiry; Encourages diversity of analysis and opinion; Promotes new research; Makes possible the testing of new or alternative hypotheses and methods of analysis; Supports studies on data collection methods and measurement; Facilitates the education of new researchers; Enables exploration of topics not envisioned by initial investigators; Permits the creation of new datasets when data from multiple sources are combined; Interoperability / technical and software standards; Comprehensive documentation of data sets and how to access them; Robust infrastructure for long-term use |
| *3* | Bezuidenhout [51] | None mentioned |
| *4* | Campbell [2] | New scientific discoveries; Stimulating economic growth, replication and validation of research; Avoidance of duplication; Consistent metadata; Data integrity |
| *5* | da Costa and Leite [47] | Standardization of data; appropriate professionals; specific funding for the management of research data; collaboration can be used as an alternative to overcome the problems of data reuse; |
| *6* | Cragin, Palmer [52] | None mentioned |
| *7* | Curty, Crowston [40] | Improving data integrity; Enhancing transparency and reproducibility of the scientific enterprise; Novel combinations of data; Opportunities for co-authorship; Data type, research discipline and traditions of data sharing; Scientist’s beliefs; Scientists’ attitudes; Experience with data use; Positive reactions to data reuse; Shortening the research process (limited time and resources); Trust in data producers; Peer pressure; Social pressure; Individual willingness; Perceptions of close colleagues; Norms; Recognition from peers; Knowledge of how to handle data; Feeling worth (e.g., many scientists feel that the time spent on data reuse is time well spent.); Demonstration of data use value |
| *8* | Enke, Thessen [10] | The ease of data accessibility |
| *9* | Fecher, Friesike [11] | Replication of research results; Application of old data in new contexts |
| *10* | Ganzevoort, van den Born [53] | None mentioned |
| *11* | Grechkin, Poon [6] | Identifying the web API for dataset access |
| *12* | Haeusermann, Greshake [18] | Accelerate research; Advance our understanding of health and disease; Value users attach to being tested |
| *13* | Harper and Kim [41] | None mentioned |
| *14* | Joo, Kim [17] | Explore new interpretations of data ; Increase the knowledge in the field; Trust in other researchers’ measurement; Academic discipline; Sector ; Policy; Context; Organizational environment; Researchers’ ability to understand open data formal training for researchers in finding, acquiring and validating data collected by others; Data documentation; Data being used enhances public trust and knowledge of the discipline; An initial large data repository to foster data sharing and reuse culture; Social norm (a researcher’s perceived belief of what other researchers think about data reuse practice); Disciplinary climate (a sense of community and openness to other researchers affiliated in the same field); Intention to reuse data; Data repository; Organizational support (any possible assistance available that researchers could acquire from their affiliated institutions or organizations, particularly technical or human help); Attitude (perceived usefulness) |
| *15* | Kim and Adler [42] | None mentioned |
| *16* | Kim and Yoon [43] | Research climate; Availability of data repositories; Perceived (data) usefulness; Availability of internal resources; Intention to reuse data; Education and institutional support (concerns about misuse of data); Technical support to ease the process (specialized software or programs); Human resource for question (advisors, data reuser groups, data producers) |
| *17* | Mooney and Newton [13] | The possibility to cite and attribute datasets, to foster a scholarly communication system that allows for identification, retrieval, and attribution of research data; Appropriate information about metadata |
| *18* | Piwowar and Vision [9] | Raw data can be used to explore related or new hypotheses, particularly when combined with other publicly available data sets |
| *19* | Piwowar, Day [8] | Explore related or new hypotheses, particularly when combined with other publicly available data sets |
| *20* | Raffaghelli and Manca [54] | Findability of the data; digital identifiers; provision of sufficient metadata; indexation as searchable resources; data exchange via a standardized communication protocol; interoperability; accurate and relevant attributes of metadata |
| *21* | Sá and Grieco [1] | None mentioned |
| *22* | Sayogo and Pardo [49] | Efficiently create more opportunities without the burden of data collection and repetition of efforts |
| *23* | Schmidt, Gemeinholzer [55] | None mentioned |
| *24* | Tenopir, Allard [56] | Added descriptive metadata to datasets |
| *25* | Wallis, Rolando [57] | For accessing the registries - catalogs of datasets that allow researchers to indicate the existence of data without going through the process of adding their data to a repository; For accessing social surveys |
| *26* | Yoon [58] | Relevance and ease of use; positive past experiences; credible information availability; study's funding sources; reputation and colleagues' recommendations; trust in the competence of the original investigator(s) (e.g. the original investigators’ membership in a Community of Practice; appropriate educational training of the original investigator); existing evaluations of the data (e.g. many existing publications using the same data; large number of times the data has been reused and cited); good intentions and ethics of the original study that produced the data (e.g. no commercial interests of the funder of the data; no apparent conflict of interest); emotional connections/ interpersonal relations with the original investigators; positive first impressions; data validity; data management; good documentation (detailed information about methodology, measurements); transparent and honest attitudes of the original investigators |
| *27* | Yoon and Kim [44] | Perceived usefulness; Considered data reuse a prevalent research practice in their research communities; Believe data reuse is good; The availability of a data repository |
| *28* | Zenk-Möltgen, Akdeniz [45] | None mentioned |
| *29* | Zimmerman [59] | Knowledge gained through disciplinary training; data-gathering experiences; familiarity with particular (comparable) types of data and areas of research and research trends; knowing that the data is available; specific knowledge about who is working in what areas; data meeting standards of scientific practice related to objectivity and representativeness; |
| *30* | Zuiderwijk [19] | Obtaining new insights; Facilitating conditions: an open data infrastructure |
| *31* | Zuiderwijk and Cligge [46] | High perceived usefulness; expectancy that effort requirements will be low; high social influence (e.g. from colleagues); high level of trust |
| *32* | Zuiderwijk and Spiers [48] | Researcher’s background; facilitating conditions, expected performance, social and affiliation factors; effort; experience; Limited resources encourages collaboration; Positive experience with open data re-use, expected performance: access to more data and obtaining new insights, being aware of the state of the art and not reinventing the wheel, feedback on the need for certain data and facilities |
