# Supplementary material for: What drives and inhibits researchers to share and use open research data? A systematic literature review to analyze factors influencing open research data adoption
Source: PLoS One. 2020 Sep 18;15(9):e0239283. doi: 10.1371/journal.pone.0239283 (PMC7500699; doi:10.1371/journal.pone.0239283)
Supplement: S5 Table — (DOCX) [file pone.0239283.s005.docx]

**S5 Table. Overview of inhibitors for using open research data by researchers, identified in the 32 studies included in our Systematic Literature Review.**

| ***No.*** | **Source** | **Factors inhibiting researchers to use open research data** |
| --- | --- | --- |
| *1* | Arza and Fressoli [4] | Costs associated to training potential data users |
| *2* | Arzberger, Schroeder [50] | Restrictions on use; Data quality (trust that data are what they purport to be); Legal restrictions (national security, privacy, trade secrets) |
| *3* | Bezuidenhout [51] | None mentioned |
| *4* | Campbell [2] | Data not accessible; Data are not findable among hundreds of data repositories; Unclear use conditions / unclear what ‘openness’ means (large variety of licenses); Varying policies on access and re-use across countries; Lack of interoperability; Data sensitivity; Inconsistent or lacking metadata |
| *5* | da Costa and Leite [47] | Lack of the necessary infrastructure for quick data analysis; issues with understanding the context of the original research and, especially, how the data were processed |
| *6* | Cragin, Palmer [52] | None mentioned |
| *7* | Curty, Crowston [40] | Difficulty to discover available and relevant data; Inability to discern dataset content and hence suitability for analysis (e.g. because of a lack of metadata); Inability to determine the quality of the data; Negative reactions to data reuse; Effort may be wasted on flawed data; Potential waste of time; Heavy reliance on the methods and techniques data producers employed to obtain, organize and code the data; Unintentionally misuse the data; The nature of data (some are easier to be reuse than others) |
| *8* | Enke, Thessen [10] | Technology-related limitation (e.g., reluctance to use online databases because of complex user inter- faces making data entry time consuming) |
| *9* | Fecher, Friesike [11] | None mentioned |
| *10* | Ganzevoort, van den Born [53] | None mentioned |
| *11* | Grechkin, Poon [6] | None mentioned |
| *12* | Haeusermann, Greshake [18] | Technical, legal, and ethical bottlenecks; Lack of harmonization of data formats, processing, analysis and data transfer |
| *13* | Harper and Kim [41] | None mentioned |
| *14* | Joo, Kim [17] | Investment of time and resources; The difficulty finding or accessing reusable data, difficulty integrating data, and the possible misinterpretation of data; Attitude (perceived concern); Potential anxiety that would be caused by data reuse activities (e.g. worry that researchers would misinterpret the data or cause an infringement problem) |
| *15* | Kim and Adler [42] | None mentioned |
| *16* | Kim and Yoon [43] | Perceived concern; Perceived effort (not significant) (the usefulness of data is important and they are willing to make efforts to reuse data) |
| *17* | Mooney and Newton [13] | Multiplicity of data types ; Lack of awareness regarding existing standards for data citation |
| *18* | Piwowar and Vision [9] | None mentioned |
| *19* | Piwowar, Day [8] | None mentioned |
| *20* | Raffaghelli and Manca [54] | Datasets requiring proprietary software to be opened; lack of interoperability; lack of references to other qualified metadata systems; not using standardized protocols; not using well-known ontologies; lack of clear usage license; data is not machine-readable; complex skills that are required for the new approaches to data |
| *21* | Sá and Grieco [1] | None mentioned |
| *22* | Sayogo and Pardo [49] | None mentioned |
| *23* | Schmidt, Gemeinholzer [55] | Data access fee; lack of familiarity of the use of the data; data quality; varying standards about data gathering; varying data formats; issue of how to access usable citation and attribution information |
| *24* | Tenopir, Allard [56] | None mentioned |
| *25* | Wallis, Rolando [57] | Data were very difficult to interpret once separated from contextual information |
| *26* | Yoon [58] | Data quality issues, such as missing variables; errors and flaws in the data; poor documentation; negative first impressions; inappropriate management or mistakes in management; original investigators 'carelessness |
| *27* | Yoon and Kim [44] | Perceived concern; Perceived effort; Quality of reusing the data based on the context of the previous study; Data quality for social scientists: good quality, trustworthy data and avoiding data with errors |
| *28* | Zenk-Möltgen, Akdeniz [45] | None mentioned |
| *29* | Zimmerman [59] | Difficult to locate the data; the lack of approaches that offer both precision and recall when it comes to locating data for reuse; challenges related to data ownership and its affect on the easy and efficient retrieval of data or information about data; risk misinterpretation based on inappropriate use of data |
| *30* | Zuiderwijk [19] | Fragmentation of datasets: data are offered at many different places; Terminology heterogeneity: each discipline has its own terminologies which leads to heterogeneity; Search options for open datasets are limited; Searching for OGD in multiple languages is often not supported; Information overload: available data and information may become overwhelming; The lack of data about the data may hinder the adequate use of these datasets; The lack of contextual information may make it difficult to analyze and interpret the data; Open data can be reused for purposes they are not meant to be used for; Data heterogeneity; Lack of support for data analysis; Tools for using OGD are fragmented and hardly integrated; Existing open data portals barely provide visualization functionalities; Interaction related to open data use is limited; Lack of interaction support and tools; Data quality concerns and changes over time; Lack of experience with open data use; Long-term availability of the infrastructure; Concerns about privacy; Trust concerns; The availability of data |
| *31* | Zuiderwijk and Cligge [46] | Low perceived usefulness; expectancy that effort requirements will be high; low social influence (e.g. from colleagues); low level of trust |
| *32* | Zuiderwijk and Spiers [48] | Data characteristics: lack of data standards, inconsistency between datasets and limited documentation; facilitating conditions, effort: the large volume and size of the datasets and required skills to analyze them, difficulties with finding the data, too much time and effort required to re-use the data, low ease of use |
