# Supplementary material for: What drives and inhibits researchers to share and use open research data? A systematic literature review to analyze factors influencing open research data adoption
Source: PLoS One. 2020 Sep 18;15(9):e0239283. doi: 10.1371/journal.pone.0239283 (PMC7500699; doi:10.1371/journal.pone.0239283)
Supplement: S1 Fig — (DOC) [file pone.0239283.s007.doc]

**Screening**

**Included**

**Eligibility**

**Identification**

Records identified through database searching
(n = 101)

Additional records identified through other sources
(n = 35)

Records after duplicates removed
(n = 119)

Records screened
(n = 119)

Records excluded
(n = 69)

Full-text articles assessed for eligibility
(n = 50)

Full-text articles excluded, because of quality issues or lack of empirical data collection
(n = 18)

Studies included in qualitative synthesis
(n = 32)
