# Supplementary material for: What drives and inhibits researchers to share and use open research data? A systematic literature review to analyze factors influencing open research data adoption
Source: PLoS One. 2020 Sep 18;15(9):e0239283. doi: 10.1371/journal.pone.0239283 (PMC7500699; doi:10.1371/journal.pone.0239283)
Supplement: S1 File — (DOCX) [file pone.0239283.s008.docx]

**Keywords (for submission)**: Open data, research data, data sharing, data use, adoption, motivation, model, factor, drivers, inhibitors, open science, open research
